# The genomic landscape of teenage and young adult T‐cell acute lymphoblastic leukemia

**DOI:** 10.1002/cam4.4024

**Published:** 2021-06-02

**Authors:** Marcela B. Mansur, Caroline L. Furness, Sirintra Nakjang, Amir Enshaei, Donat Alpar, Sue M. Colman, Lynne Minto, Julie Irving, Beth V. Poole, Elda P. Noronha, Suvi Savola, Sameena Iqbal, John Gribben, Maria S. Pombo‐de‐Oliveira, Tony M. Ford, Mel F. Greaves, Frederik W. van Delft

**Affiliations:** ^1^ Centre for Evolution and Cancer The Institute of Cancer Research London UK; ^2^ Paediatric Haematology‐Oncology Program, Research Centre Instituto Nacional de Câncer Rio de Janeiro Brazil; ^3^ Division of Clinical Research, Research Centre Instituto Nacional de Câncer Rio de Janeiro Brazil; ^4^ Wolfson Childhood Cancer Research Centre Newcastle University Centre for Cancer Newcastle upon Tyne UK; ^5^ Bioinformatics Support Unit Faculty of Medical Sciences Newcastle University Newcastle upon Tyne UK; ^6^ HCEMM‐SE Molecular Oncohematology Research Group 1st Department of Pathology and Experimental Cancer Research Semmelweis University Budapest Hungary; ^7^ Oncogenetics MRC‐Holland Amsterdam The Netherlands; ^8^ Centre for Haemato‐Oncology Barts Cancer Institute London UK

**Keywords:** clonal selection, genomics, relapse, T‐ALL, teenagers and young adults

## Abstract

**Background:**

Treatment on risk adapted intensive pediatric protocols has improved outcome for teenagers and young adults (TYA) with T‐cell acute lymphoblastic leukemia (T‐ALL). Understanding the biology of disease in this age group and the genetic basis of relapse is a key goal as patients with relapsed/refractory disease have poor outcomes with conventional chemotherapy and novel molecular targets are required. This study examines the question of whether TYA T‐ALL has a specific biological‐molecular profile distinct from pediatric or adult T‐ALL.

**Methods:**

Genomic characterization was undertaken of a retrospective discovery cohort of 80 patients aged 15–26 years with primary or relapsed T‐ALL, using a combination of Genome‐Wide Human SNP Array 6.0, targeted gene mutation and promoter methylation analyses. Findings were confirmed by MLPA, real‐time quantitative PCR, and FISH. Whole Exome Sequencing was performed in 4 patients with matched presentation and relapse to model clonal evolution. A prevalence analysis was performed on a final data set of 1,792 individual cases to identify genetic lesions with age specific frequency patterns, including 972 pediatric (1–14 years), 439 TYA (15–24 years) and 381 adult (≥25 years) cases. These cases were extracted from 19 publications with comparable genomic data identified through a PubMed search.

**Results:**

Genomic characterization of this large cohort of TYA T‐ALL patients identified recurrent isochromosome 7q i(7q) in our discovery cohort (n = 3). Prevalence analysis did not identify any age specific genetic abnormalities. Genomic analysis of 6 pairs of matched presentation – relapsed T‐ALL established that all relapses were clonally related to the initial leukemia. Whole exome sequencing analysis revealed recurrent, targetable, mutations disrupting NOTCH, PI3K/AKT/mTOR, FLT3, NRAS as well as drug metabolism pathways.

**Conclusions:**

All genetic aberrations in TYA T‐ALL occurred with an incidence similar or intermediate to that reported in the pediatric and adult literature, demonstrating that overall TYA T‐ALL exhibits a transitional genomic profile. Analysis of matched presentation – relapse supported the hypothesis that relapse is driven by the Darwinian evolution of sub‐clones associated with drug resistance (NT5C2 and TP53 mutations) and re‐iterative mutation of known key T‐ALL drivers, including NOTCH1.

## Graphical abstract

The DNA molecular profile of teenagers and young adults (TYA) T‐cell acute lymphoblastic leukemia was compared with pediatric and adult data and revealed recurrent isochromosome 7q. All genetic aberrations in TYA T‐ALL occurred with an incidence similar or intermediate to that reported in the pediatric and adult literature, demonstrating that overall TYA T‐ALL exhibits a transitional genomic profile. Relapse is driven by the Darwinian evolution of sub‐clones present at initial diagnosis associated with drug resistance (NT5C2 and TP53 mutations) and re‐iterative mutation of known key T‐ALL drivers, including NOTCH1.

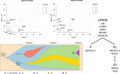

## 1 INTRODUCTION

T‐cell acute lymphoblastic leukemia (T‐ALL) is a highly aggressive hematological malignancy affecting 15% of children and 25% of adults with ALL. Current treatment protocols result in long‐term cure for 70%–80% of children and 50%–60% of adults with T‐ALL.^1^, ^2^ The outcome for teenage and young adult T‐ALL (TYA T‐ALL) has improved substantially using pediatric‐style regimens and is no longer significantly different from childhood T‐ALL.^3^ With this approach, the majority of relapses occur on treatment and are associated with abysmal outcomes.^4^

The genomic landscape of T‐ALL has been extensively explored in infants, children and adults, but no specific effort has been undertaken to explore the incidence of molecular aberrations in TYA T‐ALL. In the present study we set out to establish the existence of TYA T‐ALL specific biology through genomic characterization of an in‐house discovery cohort of 80 patients with TYA T‐ALL and to explore the origin of relapse in 6 cases with subsequent recurrence. Moreover, we performed a genetic analysis of 1792 cases of T‐ALL (including in‐house and published cases) to describe in detail age‐related incidence of genomic abnormalities. This approach will ascertain whether T‐ALL biology in TYA patients is distinct from younger and older patients and guide selection of appropriate patient populations for targeted therapies.

## 2 MATERIAL AND METHODS

### 2.1 Patients and eligibility criteria

Samples from 80 patients aged 15–26 years with primary or relapsed T‐ALL were obtained from The Royal Marsden Hospital/Institute of Cancer Research, London, UK, Haemato‐Oncology Centre, Barts Cancer Institute, London, UK, and Paediatric Haematology‐Oncology Program, Instituto Nacional de Câncer, Rio de Janeiro, Brazil (ascertainment period 2001–2012) (Table S1). The ethics committee or institutional review board of each participating center approved the study (South West Wales REC reference 12/WA/0324, CEP_INCA/CONEP#888.277). Informed consent was obtained from all subjects in accordance with the Declaration of Helsinki. In total, 104 archival bone marrow or peripheral blood samples were collected (presentation, *n* = 79; relapse, *n* = 8; matched remission, *n* = 17).

### 2.2 Identification of age specific genomic aberrations

A PubMed search was undertaken for research papers reporting DNA copy number (microarray based) and gene sequence (Sanger and/or NGS) abnormalities in T‐ALL. The authors were approached to provide age at diagnosis for individual patients. The analysis presented in this manuscript is based on 19 publications for which this information was made available (see relevant result section). Patients were separated into three age groups, namely 1–14 (pediatric), 15–24 (TYA), and over 25 (adult) years of age at diagnosis, in line with currently used UK‐based age criteria.^5^ A prevalence analysis was performed on a data set of 1792 individual cases (including in‐house cohort), including 439 TYA patients, to identify genetic lesions with age specific frequency patterns using the Fisher's exact test.

Additional methods can be found in Supporting Information.

## 3 RESULTS

### 3.1 Comprehensive genetic characterization of TYA T‐ALL

The initial genetic characterization of the in‐house discovery cohort consisted of high‐resolution copy number alteration (CNA) analysis in 69 of 80 samples. Three hundred fifty‐six CNAs were identified at diagnosis with an average of 8.1 CNA per sample (Table S2). The regions of recurrent losses and gains identified in at least 2 samples at diagnosis are shown in Table 1 and Table S3.

**TABLE 1:** Recurrent DNA copy number alterations in teenagers and young adults T‐cell acute lymphoblastic leukemia at diagnosis

Gene/region	No	Gene/region	No	Gene/region	No	Gene/region	No
*CDKN2A*	36	*PAX5*	6	*PTEN*	4	*5q34*	2
*CDKN2B*	29	*LEF1*	5	*ETV6*	4	9q34.11–34.13	2
*MLLT3*	16	*IKZF1*	5	*SUZ12*	4	*WT1*	2
*STIL*	10	*BNC2/ORF93*	5	*NR3C1*	3	*CREBBP*	2
*CASP8AP2*	8	*RB1*	5	i7(q)	3	*16q22.1*	2
*PTPRD*	7	*CTCF*	5	*DLEU7*	3		
*CDKN1B*	7	6q21	4	*NF1*	3	*MYB* gain	7
6q16.2–16.3	6	*CREB5*	4	*EBF1*	2		

Relevant to the pathogenesis of T‐ALL, this analysis identified recurrent deletion of *CDKN2A*, *CDKN2B*, *STIL*, *LEF1*, *PTEN*, 9q34.11–13, *FBXW7*, 1p33/*STIL* and gain of *MYB*.^6^, ^7^, ^8^, ^9^, ^10^, ^11^, ^12^, ^13^ The presence of the *STIL*‐*TAL1* fusion gene was confirmed by breakpoint‐specific PCR followed by Sanger sequencing in 8 cases, as was the presence of the *SET*‐*NUP214* fusion in case UPN6.^14^, ^15^, ^16^ (Figures S1–S4; Table S4).

The target gene within frequent deletions on the long arm of chromosome 6 in lymphoid malignancies remains unknown.^17^ Two common regions of deletion were identified in 9 patient samples, encompassing many putative target genes, namely *SYNCRIP*, *SNHG5*, *CASP8AP2*, *BACH2*, *EPHA7*, *CCNC* and *GRIK2*.^18^, ^19^, ^20^, ^21^, ^22^, ^23^ (Figure S5) Absence of biallelic T‐cell receptor γ locus deletion (ABD) was observed in 12 out of 51 patient samples.^24^ Chromosome 9p was the most frequent region of copy number neutral LOH affected in this cohort (*n* = 12), with all cases harboring a *CDKN2A*/*B* deletion.

### 3.2 Identification of recurrent isochromosome 7q i(7q) in TYA T‐ALL

**FIGURE 1:**
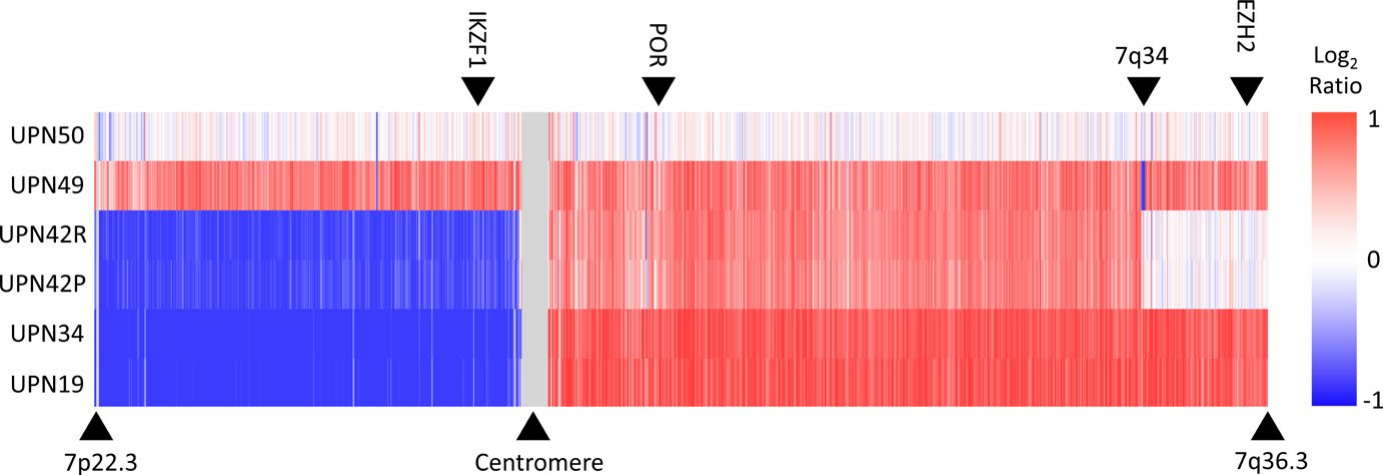
Recurrent isochromosome 7q in TYA T‐ALL. Nexus copy number analysis of chromosome 7 in 6 TYA T‐ALL samples. Analysis of UNP49 (trisomy 7) and UPN50 (disomy 7) are shown for comparative reasons. The location of MLPA probes for IKZF1, POR and EZH2 is indicated by the black arrows at the top. The color scale represents change in DNA copy number—red and blue represent gain and loss respectively. T‐ALL, T‐cell acute lymphoblastic leukemia; TYA, teenagers and young adults

Isochromosome (7q), with resultant *IKZF1* deletion, was detected in 3 cases at diagnosis (UPN19, 34 and 42). We set out to confirm the SNP array discovery of i(7q) using MLPA analyses to determine the copy number of exons in *EZH2* (7q36.1), *IKZF1* (7p12.2) and *POR* (7q11.23) (Figure 1; Figure S6). Three copies of *EZH2* were observed in UNP49 (trisomy 7) as expected. UPN42 carried 2 copies of *EZH2* explained by the diploid status of the distal part of the long arm of chromosome 7. Analysis of sample UPN19 and UPN34 showed 1 copy of *IKZF1* and 3 copies of *POR* and *EZH2* confirming the presence of i(7q), initially identified by SNP array. Interestingly, as UPN42P and UPN42R are matched diagnostic and relapse samples from the same patient, our analyses suggest that this lesion is preserved throughout disease progression.

### 3.3 Validation of identified CNAs

MLPA was performed in 48 samples confirming all previously identified DNA losses and gains. The MLPA analysis was suggestive of *LEF1* intragenic deletions in 2 samples (UPN 2, 32), which had not been detected by the initial SNP array screening (Figures S7 and S8). Exon specific copy number qPCR (exon 4, 7, 13) confirmed deletion of exon 4 in both cases (Figure S9). MLPA analysis of 4 samples (UPN 11, 26, 32, 50) was further suggestive of intragenic deletions within the *PTEN* gene also not detected by SNP array. Sanger sequencing of *PTEN* exon 7 confirmed indel mutations in 2 samples (UPN 11, 50), providing a potential explanation for these findings.^25^

### 3.4 Target gene mutation screening

Gene mutation screening was undertaken in selected genes implicated in the pathogenesis of T‐ALL (Table S5). Our study confirmed the NOTCH signaling pathway as the main mutated target in T‐ALL. At diagnosis, *NOTCH1* mutations were identified in 46/79 (58.2%) and *FBXW7* mutations in 17/79 (21.5%). *NOTCH1* mutations were found in the HD, PEST/TAD domains or both in 58.7%, 21.7% and 19.6%, respectively. Eight cases carried both *NOTCH1* and *FBXW7* mutations, and overall 55/79 (78.5%) diagnostic samples carried mutations leading to aberrant NOTCH pathway activation. *PTEN* and *IL7R* mutations were found in 12/79 (15.2%) and 5/79 (6.3%) of diagnostic cases, respectively (Figure S10). Screening of Janus kinases identified no mutations in *JAK2* exon 14. One potential new mutation (p.V651L) was observed in *JAK1*. The latter occurred in the pseudo‐kinase domain that is essential for normal kinase activity. This variation is absent from both the dbSNP and the 1000 genome project databases. Two known mutations in *TP53* (V218A, R248Q) were identified. Mutation screening of the RAS pathway identified 2 mutations in *NRAS* (G12S, Q61P), 1 mutation in *CBL*, 1 mutation in *KRAS* (G13D), and no mutations in *PTPN11* (*SHP2*, exons 3 and 13), or *FLT3* (exons 14 and 20).^26^, ^27^

### 3.5 Inactivation of the CDKN2A locus in T‐ALL

DNA copy number analysis identified *CDKN2A* deletion in 70.6% of presentation samples. Retention of wild‐type *CDKN2A* was confirmed by FISH in 4 cases (UPN 6, 11, 23, 27) (Figure S11). The *CDKN2A* locus could also be inactivated through gene mutation or promoter methylation.^28^ Promoter methylation analysis in patient samples that retained one or both gene copies of *CDKN2A* demonstrated clear methylation in only 2 out of 23 tested samples (UPN 6, 27) (Figure S12).

### 3.6 Identification of age specific genomic aberrations

**FIGURE 2:**
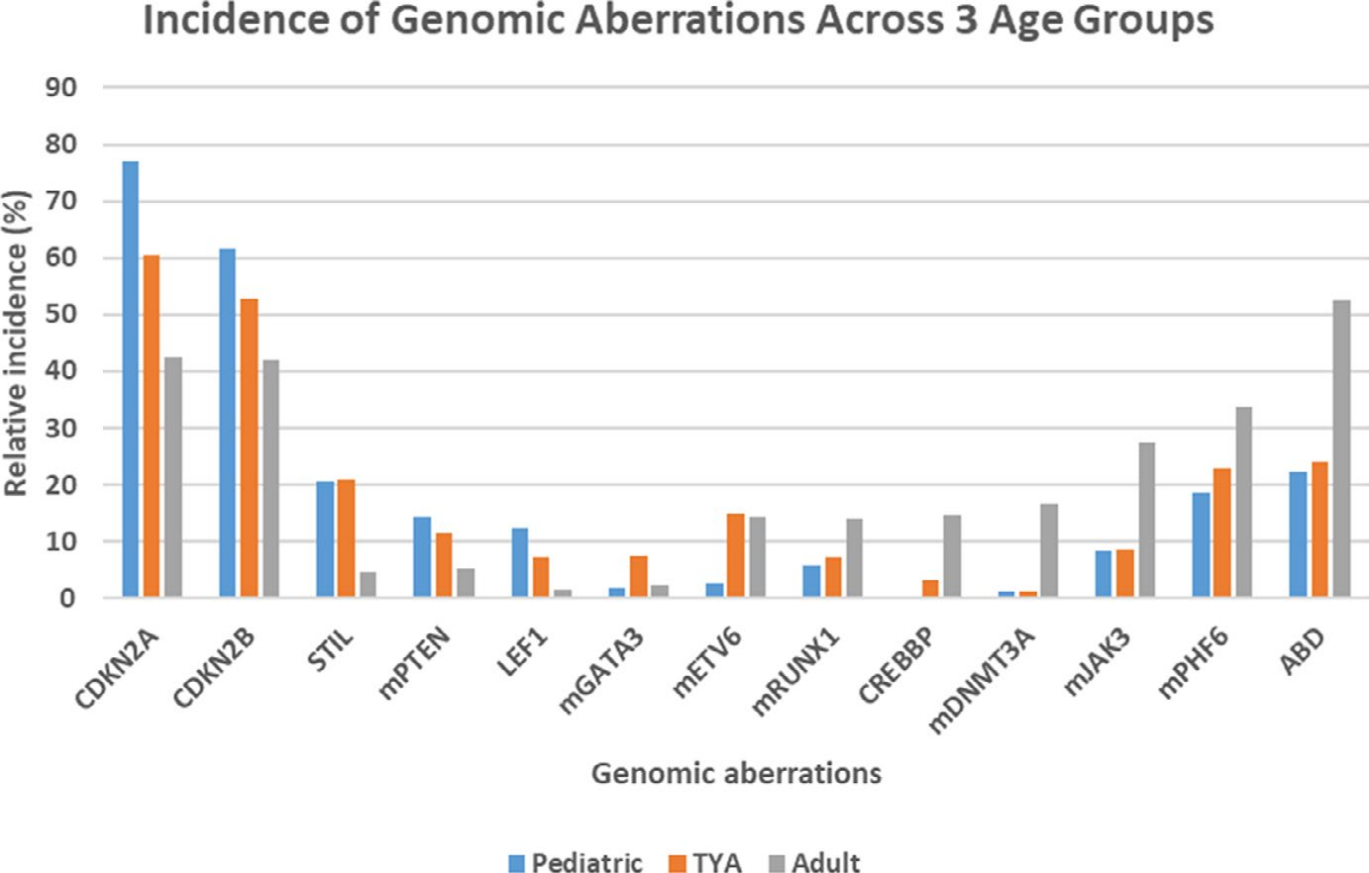
Incidence of gene abnormalities with significant enrichment in T‐ALL age groups. Bar diagrams depict the relative incidence of gene deletions and mutations (mGene) with significant enrichment in Paediatric, TYA and/or Adult patients with T‐ALL. T‐ALL, T‐cell acute lymphoblastic leukemia; TYA, teenagers and young adults

The genomic landscape of pediatric and young adult T‐ALL was recently characterized.^29^ We used this extensive data set in combination with our in‐house and other published data to perform a comprehensive incidence analysis aiming to identify any potential TYA specific genomic aberrations. Nineteen studies were identified with available age at diagnosis, DNA copy number and/or gene sequence data.^6^, ^7^, ^9^, ^10^, ^11^, ^12^, ^24^, ^29^, ^30^, ^31^, ^32^, ^33^, ^34^, ^35^, ^36^, ^37^, ^38^, ^39^, ^40^, ^41^ The total patient cohort included 1792 individual patients, who were then separated into three age groups: 1–14 (*n* = 972); 15–24 (*n* = 439) and ≥25 (*n* = 381) years of age at diagnosis. Statistical analyses were performed on 148 genes and/or gene regions to identify genetic lesions with age‐specific incidence patterns (Table S6). The incidence of *NOTCH1* mutations in the 3 age groups was 61.5%, 58.8% and 64% respectively, and was not significantly enriched in any age group. Similarly, *FBXW7* mutations were equally distributed (Table S6). The incidence of 17 abnormalities was significantly enriched or depleted in one or two of the three age groups (Figure 2).

The pediatric group was enriched for deletion of *CDKN2A*/*B* and *LEF1* as well as mutation of *PTEN*. The adult cohort was enriched for deletion of *CREBBP*, ABD, as well as mutation of *DNMT3A*, *JAK3*, *PHF6* and *RUNX1*. Deletion of *NOTCH1* and *RUNX1*, as well as mutation of *CDKN2A* and *PTPN2*, were significantly enriched in adult T‐ALL. However, the latter four abnormalities were detected in <10 cases overall, hence these results should be interpreted with caution and were removed from Figure 2.

Pediatric and TYA T‐ALL were significantly enriched for 1p33/*STIL* abnormalities, whilst TYA/adult T‐ALL were enriched for gene mutations in *ETV6* and *GATA3*. No aberration was solely enriched in our TYA cohort.

The therapeutic consequences of these different mutation patterns were explored by performing pathway analyses. Gene alterations were functionally mapped into four pathways. Using the initial 148 gene panel, two of these were significantly enriched in the pediatric group providing actionable targets, such as PI3K‐AKT and RAS. We observed a trend for increased incidence of JAK‐STAT and NOTCH pathway abnormal activation, particularly in the pediatric group (Table S7).

### 3.7 The genetics of relapsed TYA T‐ALL

The DNA copy number analysis of 8 in‐house relapse samples showed alterations of known drivers, such as loss of *CDKN2A*/*B*, *MLLT3*, *PTEN*, 6q16.2–16.3, 6q21, *IKZF1*, *NR3C1*, *CASP8AP2*, *TOX*, *PAX5*, 9q34.11–34.13, *RAG1*/*2*, *WT1*, *RB1*, *DLEU7*, *CTCF*, *NT5C3* and gain of 10p (Table S3). When compared with matched presentation, additional loss of 6q14.3–21 (including *CASP8AP2*) and *MLLT3*/*CDKN2A*/*B* was observed in one patient each at relapse.

Whole exome sequencing (WES) was undertaken in 4 in‐house cases of TYA T‐ALL with available matched presentation, remission and relapse samples. Overall, 739 somatic Single Nucleotide Variation (SNVs) and 213 insertion and/or deletions (indels) were identified. Three hundred and eight (41.7%) of the somatic mutations were predicted to be protein altering, with the majority being missense mutations (34.2%); the rest were frameshift indels (2.5%), in‐frame indels (2.3%) and nonsense (2.3%) mutations (Table S8). Protein‐altering abnormalities occurred across 308 genes (Table S9). On average, each sample contained 32.4 somatic protein‐altering SNVs and indels. In our case series we observed non‐significant increase in average number of protein‐altering mutations with 28.5 (range 24–34) at presentation and 31.3 (19–38) at relapse.

**FIGURE 3:**
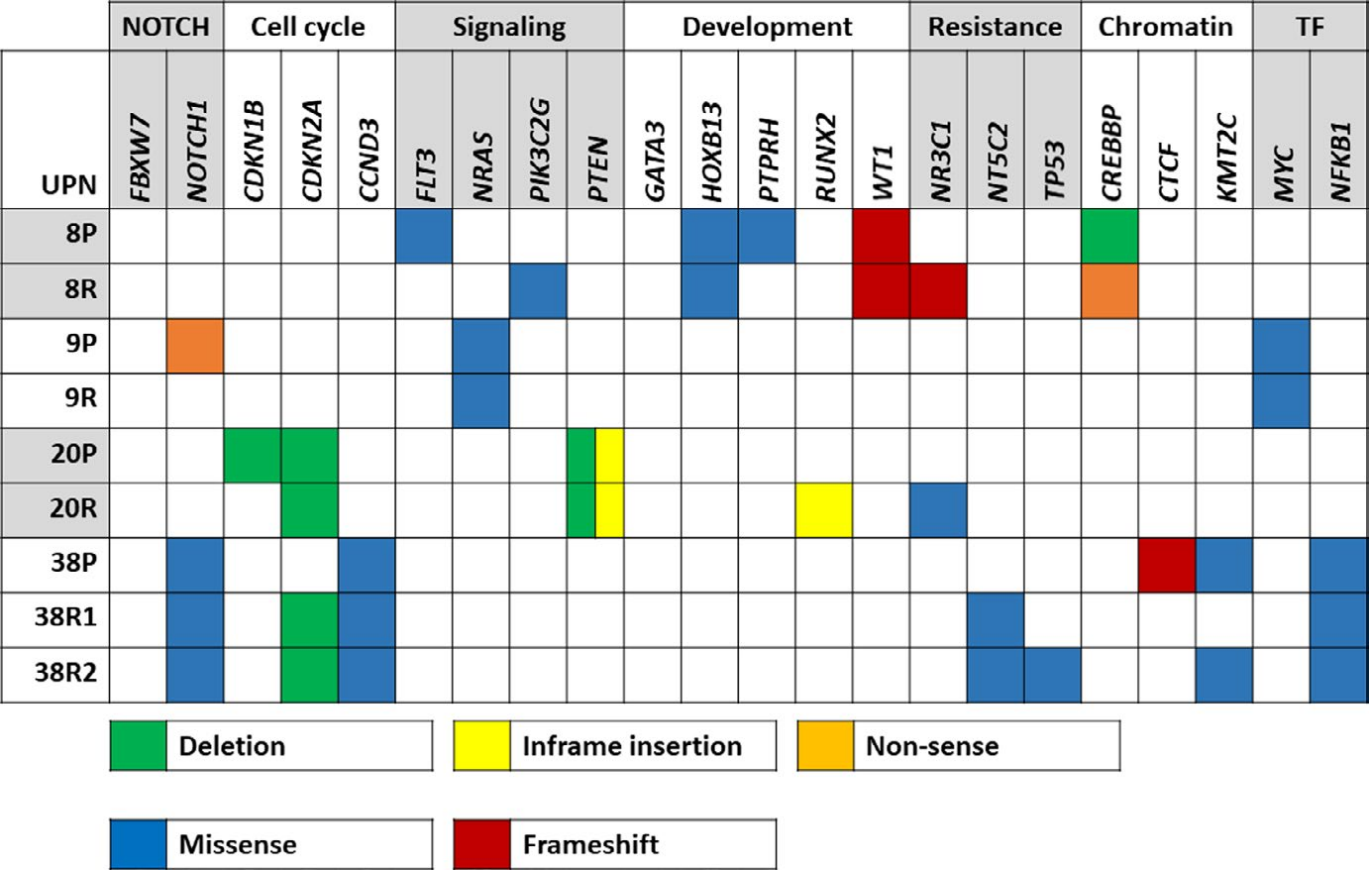
Heatmap of recurrently targeted pathways in matched presentation and relapsed TYA T‐ALL. Heatmap of selected genes affecting cellular pathways in T‐ALL. Validated and presumed protein‐altering mutations are shown. Green boxes indicate deletions; blue boxes missense mutations; yellow boxes in‐frame insertions; red boxes frameshift mutations; orange boxes nonsense mutations. T‐ALL, T‐cell acute lymphoblastic leukemia; TF, transcription factor; TYA, teenagers and young adults

Whole exome sequencing analyses revealed recurrent mutations affecting the NOTCH and PI3K/AKT/mTOR pathways (Figure 3). Furthermore, mutations in *FLT3*, *NRAS*, *MYC*, *NR3C1*, *NT5C2* and *CREBBP* suggest disruption of relevant oncogenic pathways and drug metabolism. One case carried an inactivating mutation of Protein Tyrosine Phosphatase, Receptor Type, H (PTPRH) which is predicted to provide additional growth and survival signals through activation of LAT and ZAP70, two kinases involved in pre T‐cell receptor signaling.^42^, ^43^

### 3.8 Origin of relapse in TYA T‐ALL

Previous studies have demonstrated that a clonal relationship is evident in most cases of relapsed T‐ALL.^8^, ^30^ We used SciClone to analyze variant allele frequencies of somatic mutations, in combination with CNA data, to infer clonal architecture and pattern of tumor evolution under treatment pressure. In all 6 in‐house matched cases, the relapse clone shared at least a single genetic aberration with the presentation leukemia. The molecular composition of the relapse clone either fully resembled the diagnostic clone (UPN9, 42) or acquired additional mutations (UPN20, 21, 38). In case UPN8, the presentation and relapse leukemia shared a somatically acquired *WT1* mutation. Strikingly, copy number gains and losses changed completely between the 2 time points (Figure S13).

Clinically, these 6 patients were treated with a variety of historic treatment protocols. All achieved complete remission after initial induction therapy. The relapses in our cohort occurred after a median remission duration of 1.0 year (0.3–2.4 years). Four patients relapsed very early (<18 months from diagnosis). Case UPN8, in which relapse was likely driven by an ancestral clone, exhibited the longest time to relapse (2.4 years). Despite 3 patients receiving a bone marrow transplantation, none of the 6 relapsed patients is currently alive.

**FIGURE 4:**
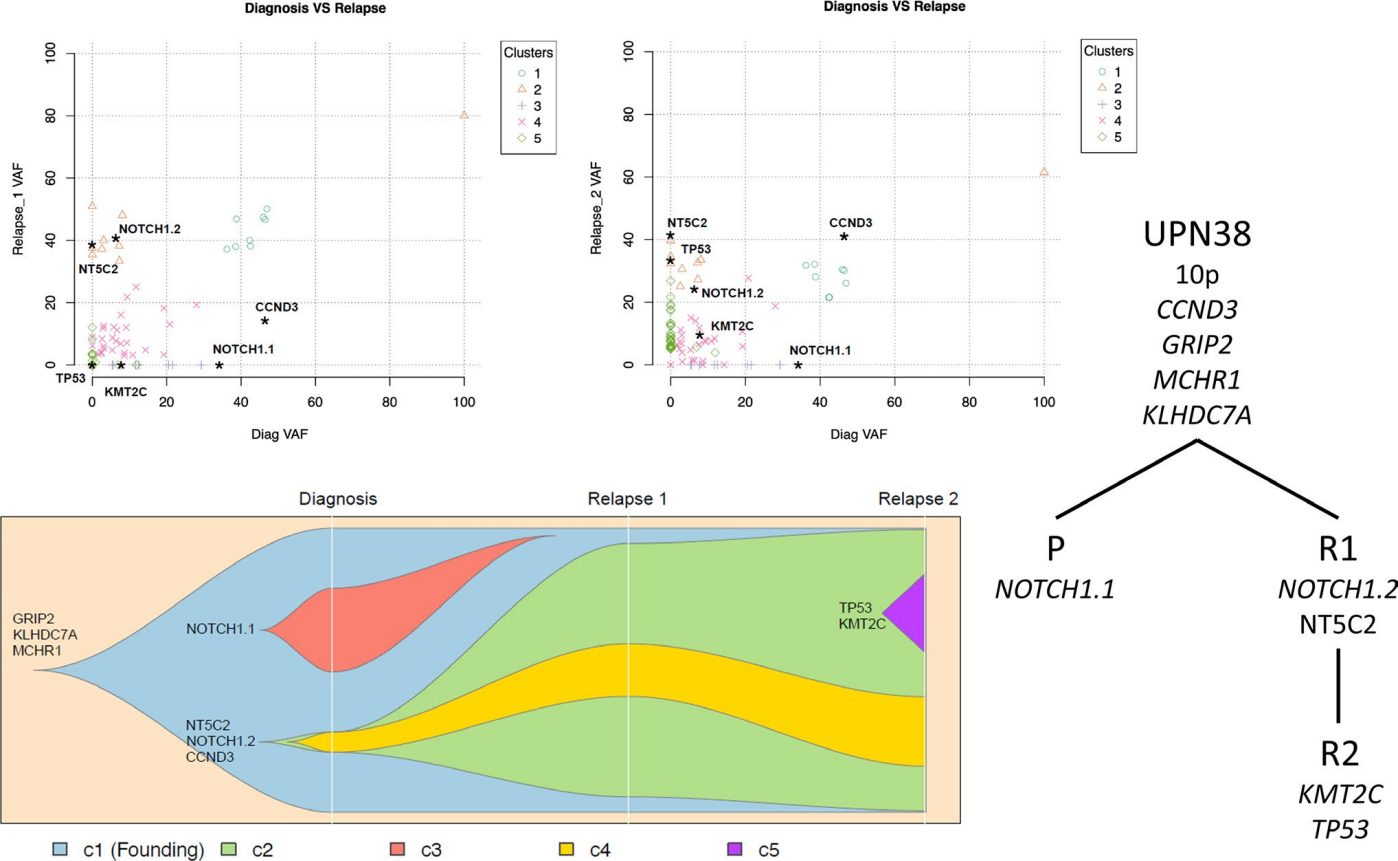
Comparative genetic composition of UPN38 at presentation and subsequent relapses. Top, variant allele frequency (VAF) of missense mutations in presentation, relapse 1 and 2. Bottom, Inferred clonal evolution from a single hematopoietic stem cell to presentation (P), relapse 1 (R1) and relapse 2 (R2). Phylogenetic tree depicting shared and acquired gene mutations

UNP38 represents an interesting case with 2 subsequent relapses (Figure 4). Presentation and both relapse samples were characterized by gain of 10p and an acquired missense mutation in *CCND3*. The *CCND3* mutation (K268T) was found in a mutational hotspot in exon 5.^44^ *CCND3* mutations were shown to increase cell proliferation rate, further enhanced by *CDKN2A* deletions at relapse.^44^ The diagnostic sample further harbored 2 distinct *NOTCH1* mutations with only one of these mutations present at both relapses, indicating subclonal evolution underlying relapse. Inactivating mutations in *KMT2C* (at presentation and second relapse) have previously been described in refractory diffuse large B‐cell lymphoma and suggest a role for chromatin remodeling and histone modulation.^45^ Both relapses carried an *NT5C2* missense mutation suggesting escape of a drug resistant clone, which ultimately also acquired or selected for a *TP53* mutation.

## 4 DISCUSSION

Genome‐wide DNA copy number and target gene mutation analyses in our in‐house TYA cohort confirmed the plethora of genetic markers known to play a role in T‐ALL.^46^, ^47^ The average number of CNAs identified was similar as reported for pediatric (3.3–7.1) and adult (6–6.8) T‐ALL.^7^, ^10^, ^11^, ^34^ Remarkably, 3 out of 51 (5.8%) presentation cases were characterized by i(7q). Although this abnormality has previously been described in T‐ALL, it mostly occurs in hyperdiploid B‐lineage ALL.^48^ Moreover, i(7q) is a typical feature of Hepato Splenic T‐cell Lymphoma.^49^ With an estimated incidence of i(7q) in ALL around 0.9%, our analysis showed an increased incidence in TYA T‐ALL.^48^ Isochromosome 7q was not reported or observed in other TYA T‐ALL cases (*n* = 388) in our extended analysis. The incidence reported in our discovery cohort might thus be an underestimate and can only be fully appreciated if genome wide copy number DNA analysis are performed.

We have previously established that the rare subtype of infant T‐ALL has a distinctive genomic profile with *MLF1* and *KMT2A* gene abnormalities and a relatively low incidence of *CDKN2A* deletions.^50^ Our current statistical analysis of nearly 1800 cases (in‐house and published) revealed uneven distribution of only 17 out of 148 genes/gene regions tested, underlining broad biological similarities in T‐ALL biology across the age groups evaluated (pediatric, TYA and adults) (Table S10). No single DNA abnormality was specific for the TYA age group.

Our dataset confirmed a trend for the decreasing occurrence of *LEF1*, *PTEN*, *RPL10*, *STIL*, *TLX3* and increase in *CNOT3*, *CREBBP*, *DNMT3A*, *JAK1*, *PHF6*, *PICALM*‐*MLLT10*, *TLX1* abnormalities with older age.^12^, ^43^, ^51^, ^52^ Phenotyping data were unfortunately not available for our TYA cohort. We propose however that the increased incidence of the early T‐cell precursor‐ALL phenotype in adults explains the observed enrichment in ABD and mutations in *ETV6*, *GATA3*, *RUNX1* and *JAK3* in adult T‐ALL.^11^, ^24^, ^29^, ^37^, ^41^, ^53^, ^54^

The genetic basis of relapsed T‐ALL was explored using SNP arrays and/or WES. Previous work has highlighted the lack of acquisition of additional CNAs in relapsed childhood T‐ALL, in contrast with relapsed BCP‐ALL.^30^ This was confirmed in our study of 6 matched cases with an average number of CNAs of 7.6 at diagnosis and relapse. Previously, exome sequencing of T‐ALL had identified an average of 8.2 and 21.0 protein‐altering mutations in pediatric and adult patients, respectively.^43^ The higher incidence of somatic mutations in our data set could reflect the true mutation rate in TYA T‐ALL. However, it is conceivable that the difference in mutation rate may be due to the small cohort analyzed and the difference in bioinformatic workflows used. WES analyses of matched pediatric T‐ALL at diagnosis and relapse has previously shown an increase in SNVs and small indels at relapse, which we could not corroborate.^8^

The TYA genomic landscape presents ample scope for introduction of targeted therapies at relapse, as exemplified by aberrations in NOTCH, PI3K/AKT/mTOR, IL7R/JAK/STAT and RAS pathways. Gene mutations associated with drug resistance (*NR3C1*, *NT5C2*, *CREBBP* and *TP53*) were gained at relapse, suggesting evolutionary selection of sub‐clones endowed with survival advantage.^55^, ^56^ Encouragingly, synergy between small molecule inhibitors and dexamethasone has been shown to overcome glucocorticoid resistance in T‐ALL.^57^, ^58^, ^59^, ^60^

Notably, several mutations (*NOTCH1*, *FLT3*, *PTEN*) were ‘lost’ at relapse, indicating they might represent non‐founder or subclonal mutations.^15^, ^30^, ^61^ This underlined our previous data, which highlighted at single cell level that *NOTCH1* and *PTEN* mutations were subclonal and demonstrated convergent evolution.^15^

Our study was limited to retrospective analysis of historic samples with incomplete information on immunophenotyping, protocol and outcomes. This impeded identification of prognostic biomarkers. This further precluded complete genetic annotation with RNA sequencing to identify oncogene expression and co‐occurrence of mutations in individual samples. Such an approach would have allowed further comparison with pediatric T‐ALL, whilst such data in adult T‐ALL are mostly lacking.

In conclusion, our study has described for the first time the genomic landscape of TYA T‐ALL and identified a relatively high incidence of isochromosome 7q. Our observations support the existence of age‐related biological differences between infants, children, TYA and adult patients. WES analyses suggested the major route to relapse is selection of drug resistant clones under therapy pressure and identified bona fide drug targets. Although these observations will inform the design of future salvage therapies, they should not be seen in isolation and require integration with functional profiling of relapse cells incorporating the protective role of the bone marrow niche.

## CONFLICT OF INTEREST

The authors declare no conflict of interest.

## ETHICAL APPROVAL

The ethics committee or institutional review board of each participating center approved the study (South West Wales REC reference 12/WA/0324, CEP_INCA/CONEP#888.277). Informed consent was obtained from all subjects in accordance with the Declaration of Helsinki.

## Supporting information

Supplementary Material

## Data Availability

The raw data CEL and fastq files are available upon request and will be accessible through the Newcastle University data repository (data.NCL).
