# Supplementary material for: The genomic landscape of teenage and young adult T‐cell acute lymphoblastic leukemia
Source: Cancer Med. 2021 Jun 2;10(14):4864–73. doi: 10.1002/cam4.4024 (PMC8290240; doi:10.1002/cam4.4024)
Supplement: Supplementary file 1 — Supplementary Material [file CAM4-10-4864-s001.zip › cam44024-sup-0001-Supinfo.docx]

**Supporting information: “The Genomic Landscape of Teenage and Young Adult T-cell Acute Lymphoblastic Leukemia”**

**Data S1. Methods**

*Nucleic acid extraction*

DNA and RNA were extracted simultaneously from buffy coat using the AllPrep DNA/RNA Mini Kit (Qiagen, Crawley, UK) and quantified using the NanoDrop 1000 Spectrophotometer (Thermo Scientific, Wilmington, DE, USA). The quality of total RNA was verified using Agilent RNA 6000 Nano Kit (Agilent Technologies, Santa Clara, CA, USA).

*Genome mapping analysis*

Mapping analysis was performed with 500ng of tumor and germline DNA from each patient as previously described.[^1^](#_ENREF_1) DNA was prepared according to the manufacturer's instructions with the use of the Genome-Wide Human SNP Array 6.0 (Affymetrix, Santa Clara, CA, USA). The CEL files are available upon request.

*CNA and Loss-Of-Heterozygosity (LOH) analysis*

SNP genotypes were obtained with the use of the BRLMM algorithm in Affymetrix Genotyping Console 4.0 software. Samples were analyzed with dCHIP 2009 and CNAG 3.3.0.1 (beta).[^2^](#_ENREF_2) The position of regions of LOH and gain were identified with the University of California Santa Cruz Genome Browser (NCBI36/hg18, March 2006 Assembly).

*Multiplex ligation-dependent probe amplification (MLPA)*

The SALSA MLPA P383 T-ALL and P335 ALL-IKZF1 probe mixes (MRC-Holland, Amsterdam, Netherlands) were used to assess gene copy number of different chromosomal regions according to the manufacturer's instructions. Normal diploid controls were included for normalization.

*Exon specific copy number quantitative real-time PCR (qPCR)*

The genomic copy number of exons 4, 7 and 13 of the *LEF1* gene was investigated by quantitative real-time PCR (qPCR) using the following commercial exon specific TaqMan® DNA copy number assays: Hs02369478_cn (exon 4), Hs01540634_cn (exon 7) and Hs02543358_cn (exon 13) ([Thermo Fisher Scientific](http://www.thermofisher.com/etc/countries/united-kingdom.html), Paisley, UK). Human *RNaseP* was used as the reference gene ([Thermo Fisher Scientific](http://www.thermofisher.com/etc/countries/united-kingdom.html), Paisley, UK). The qPCR reactions were performed in triplicate on a 7900HT Fast Real-Time PCR System ([Thermo Fisher Scientific](http://www.thermofisher.com/etc/countries/united-kingdom.html), Paisley, UK) as per the manufacturer's protocol. For the relative quantification analyses, 2 normal diploid controls were used as reference DNAs with a known diploid status for *LEF1* copies status. Per reaction 10ng of genomic DNA was used. Alongside 4 diagnostic DNA samples, the 2 normal diploid and 1 no template controls were included. Data evaluation was performed with the ABI 7500 SDS software. A fold change ratio of target gene/reference gene <0.7 was considered indicative of gene loss.

*Targeted gene mutation screening*

*NOTCH1*, *FBXW7*, *PTEN* and *IL7R* mutations were all analyzed by conventional PCR screening using genomic DNA, followed by PCR purification and subsequent Sanger sequencing using the BigDye Terminator v3.1 kit ([Thermo Fisher Scientific](http://www.thermofisher.com/etc/countries/united-kingdom.html), Paisley, UK). All procedures performed for the target mutation screening were previously described elsewhere.[^3-7^](#_ENREF_3)

For *NOTCH1* the heterodimerization (HD) domain (encoded by exons 26 and 27) and polypeptide enriched in proline, glutamate, serine and threonine (PEST) / transcriptional activation (TAD) domains (encoded by exon 34) were targets for the mutational screening. To evaluate the mutational status of *FBXW7*, we screened exons 9 and 10, previously reported to be the most frequently mutated spots.[^5^](#_ENREF_5)^,^[^8^](#_ENREF_8) *PTEN* exons 1 and 7 and *IL7R* exon 6 were amplified and analyzed accordingly.

*Targeted gene mutation screening in RAS pathway*

*NRAS* exons 1 and 2, *KRAS* exon 1, *CBL* exons 8 and 9, *PTPN11* (*SHP2*) exons 3 and 13, *FLT3* exon 14 and 20, *JAK1* exon 14 and *JAK2* exon 14 were screened for mutations using denaturing high performance liquid chromatography (dHPLC) on a Transgenomic WAVE machine using genomic DNA or in some cases whole genome amplified DNA. Amplicons were analyzed before and after spiking with known wild type product to allow detection of homozygous mutations. Primer sequences and dHPLC conditions are available on request. Direct sequencing and PCR product cloning were performed using standard techniques.[^9^](#_ENREF_9)

*TP53 mutation screening*

Capillary electrophoresis single-strand conformation analysis (CE-SSCA) was used to detect mutations in exons 4-9 of the *TP53* gene. Any mutations identified were further characterized by Sanger sequencing as previously described.[^10^](#_ENREF_10)

*Fluorescence in situ hybridization - FISH*

Single-cell suspensions were fixed in 3:1 methanol-acetic acid using standard cytogenetic methods.[^11^](#_ENREF_11) Interphase FISH for selected oncogenes and CNAs was performed using either commercial Cytocell probes (Cambridge, UK) (*TLX1, CDKN2A*) or in-house probes (*TLX3, CDKN2A*) as previously described.[^11-13^](#_ENREF_11) (Table SX) For in-house probes Bacterial Artificial Chromosome (BAC) and fosmids for selected genes were obtained from the BACPAC Resource Centre, Children's Hospital, Oakland Research Institute (<http://bacpac.chori.org>) or Invitrogen ([Thermo Fisher Scientific](http://www.thermofisher.com/etc/countries/united-kingdom.html), Paisley, UK). These were labelled with SpectrumOrange^TM^, SpectrumRed^TM^ or SpectrumGreen^TM^ fluorochromes (Vysis, Abbott Laboratories) or nick translated with biotin-16-dUTP (Roche Diagnostics), and hybridized in combination as previously described.[^11^](#_ENREF_11) Hybridization and washes were performed according to the Vysis protocol, with biotinylated probes detected by a single layer of [Cy®5-Streptavidin](http://www.sigmaaldrich.com/catalog/product/sigma/gepa45001) (GE Healthcare). Fluorescent signals were viewed with a Zeiss Axioskop fluorescence microscope equipped with filters for DAPI, FITC / Spectrum Orange, Red, Green and Cy5. Images were captured and analyzed using SmartCapture X software (Digital Scientific, Cambridge, UK).

*Promoter methylation status of CDKN2A*

Methylation specific PCR (MSP) of the *CDKN2A* promoter region was performed as described previously.[^14^](#_ENREF_14) Briefly, genomic DNA underwent sodium bisulphite modification using EpiTect Bisulfite Kit according to the manufacturer’s instructions (QIAgen). MSP was performed in 25 patient samples with hemizygous or no *CDKN2A* gene deletion and 2 control cell lines (HL60 cell line – positive control for unmethylated promoter; RAJI cell line – positive control methylated promoter).[^15^](#_ENREF_15) The PCR products were visualized with 2% agarose gel stained with ethidium bromide and confirmed by Sanger sequencing.

*Confirmation of STIL-TAL1 fusion*

DNA from 8 patients with *STIL-TAL1* fusion, as identified by genome mapping analysis, was subjected to PCR using the 5’ *STIL* and 3’ type 1 *TAL1* primers to confirm gene fusion as previously described.[^16^](#_ENREF_16) The PCR products were visualized with 1.5% agarose gel stained with ethidium bromide, purified and analyzed by Sanger sequencing. (Fig S2 and Table SIV)

*Oncogene expression in T-ALL*

The gene expression of the oncogenes *TLX1* (Hs00271457_m1) and *TLX3* (Hs01099063_g1) was established using a TaqMan® qPCR approach and *β2-microglobulin* (Hs99999907_m1) was used as reference gene. cDNA was synthesized using SuperScript® III First-Strand Synthesis System (Life Technologies, Paisley, UK) as per manufacturer’s protocol. The PCR reactions were performed in triplicate on the ABI 7900 PCR machine ([Thermo Fisher Scientific](http://www.thermofisher.com/etc/countries/united-kingdom.html), Paisley, UK); with the following cycling conditions 50°C for 2 min, 95°C for 10 min, 95°C for 15 s and 60°C for 1 min for 40 cycles. Relative gene expression for *TLX1* and *TLX3* is shown in Fig S14 and S16. FISH confirmation of representative patients is shown in Fig S15 and S17.

*Identification of fusion transcripts*

Screening for *SET-NUP214* and *PICALM-MLLT10* fusions was performed as previously described.[^17^](#_ENREF_17)^,^[^18^](#_ENREF_18) PCR reactions were performed using 40ng cDNA (8 ng/μL), 10pmol primers, 10nmol dNTPs, 1.5mM MgCl_2_, 1.25U ampliTaq gold (Applied Biosystems, Foster City, CA) in 10× PCR buffer II in a total volume of 50μL. After the initial denaturation at 94°C for 10 minutes, PCR was performed for 39 cycles of 95°C for 15 seconds, 60°C for 1 minute, and 68°C for 3 minutes. The PCR products were visualized on a 1% agarose gel stained with ethidium bromide, purified and analyzed by Sanger sequencing. (Fig S4 and S18)

*Whole exome sequencing (WES)*

Genomic DNA was whole-exome sequenced (Genomatix, Munich, Germany). Exome capture was performed using the Agilent SureSelectXT Human All Exon v4 kit following manufacturer’s procedures (Agilent, Santa Clara, CA, USA) and sequenced with Illumina 100bp paired end sequencing (protocol v1.2) on an Illumina HiSeq 2000. Raw reads were aligned to human reference genome (hg19) using Burrows–Wheeler Aligner (BWA) 0.6.2 [^19^](#_ENREF_19) and were processed using the Genome Analysis Toolkit (GATK, v3.3.0) best practices recommended workflow for variant discovery analysis.[^20-22^](#_ENREF_20) MuTect (v1.1.7), MuTect2, and VarScan 2 (v2.3.7) were used to identify somatic variants for each matched pair samples.[^23^](#_ENREF_23)^,^[^24^](#_ENREF_24) Variants were annotated using Ensembl Variant Effect Predictor (VEP)(Tables SVIII, IX, XI) summarize genome coverage and somatic variants affecting coding regions).[^25^](#_ENREF_25) Identified variants were visually inspected using Integrative Genomics Viewer (IGV v2.3). Several gene mutations identified by WES analysis in *NOTCH1*, *PTEN* and *NR3C1* were independently validated using Sanger sequencing. The raw data fastq are available upon request. Subclonal populations were identified using SciClone.[^26^](#_ENREF_26) ClonEvol and FishPlot were used to infer clonal evolution and to display changes in clonal structure over time.[^27^](#_ENREF_27)^,^[^28^](#_ENREF_28)

**Data S2. Supplementary figures**


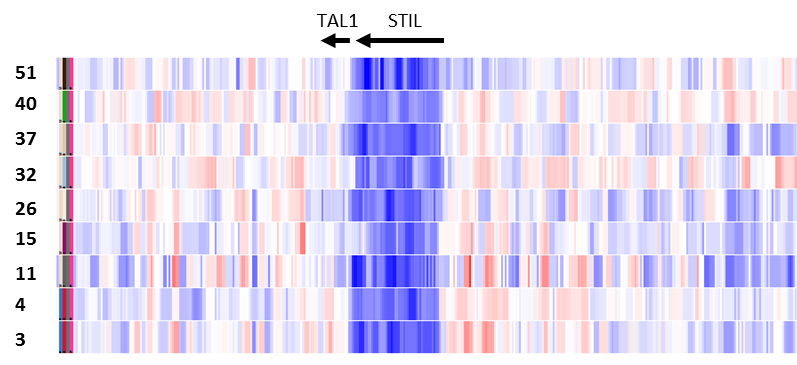


**Fig S1.** DNA copy number of *STIL*. dCHIP DNA copy number analysis of chromosomal region 1p33. Red indicates gain and blue indicates loss of DNA. The recurrent deletion in 9 patient samples represents almost complete deletion of the *STIL* gene leading to the formation of the fusion gene *STIL-TAL1*.


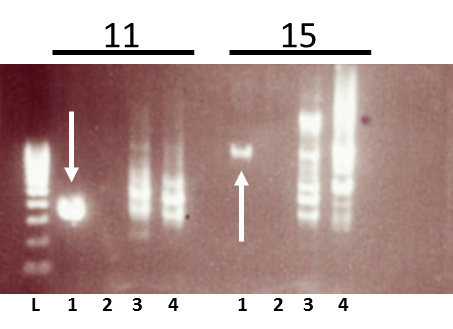


**Fig S2.** Electrophoresis of *STIL* deletion fusion regions on 1% agarose gel for samples UPN11 and UPN15. L = 100bp ladder (Bioline). Numbers 1 – 4 refer to two separate PCR reactions using 5’ *STIL* and 3’ type 1-4 *TAL1* primers.[^16^](#_ENREF_16) The white arrows indicate DNA bands subjected to conventional Sanger sequencing.


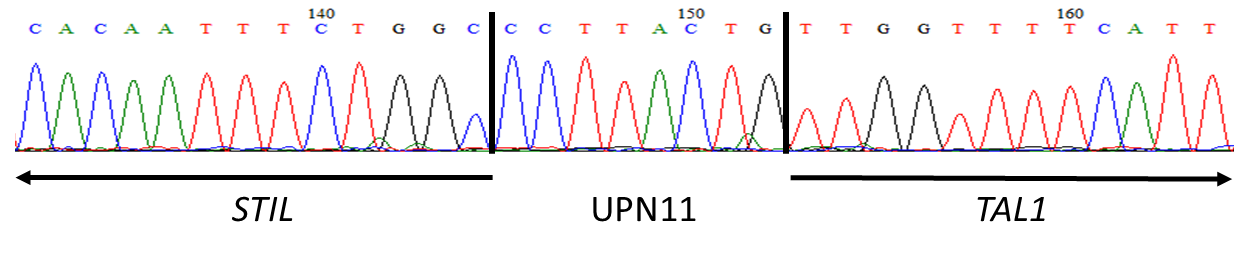


**Fig S3.** Electropherogram of sample UPN11 with interspersed nucleotides indicating the fusion region between *STIL* and *TAL1*.


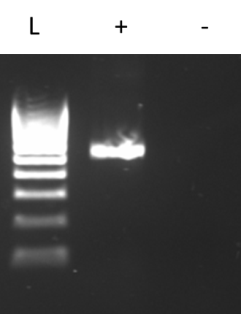


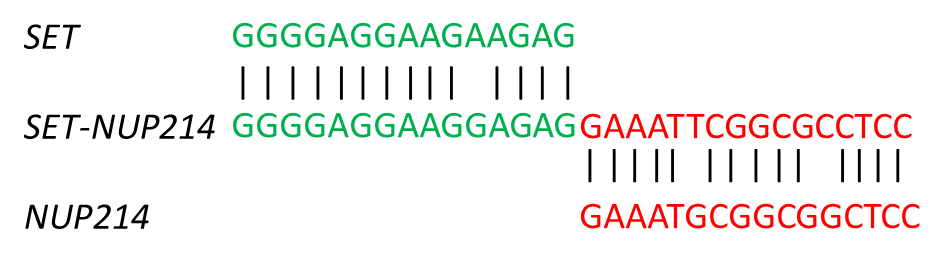


**Fig S4*.*** *SET-NUP214* PCR analysis. DNA sample from UPN6 demonstrated an 800bp product with Sanger sequencing demonstrating a gene fusion of *SET* exon 7 with *NUP214* exon 18. L = 100bp ladder (Bioline), + indicates PCR reaction with gDNA, - indicates negative control.


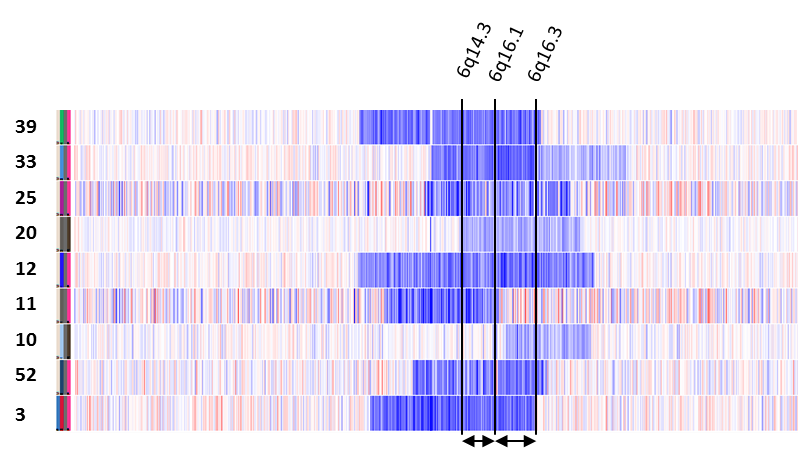


**Fig S5.** DNA copy number of 6q. dCHIP DNA copy number analysis of chromosomal region 6q. Red indicates gain and blue indicates loss of DNA. Two common regions of deletion, represented by the black arrows, were identified in 9 patient samples. Region 1 (6q14.3-q16.1; 85,203,739-95,846,751 base pairs) and region 2 (6q16.1-q16.3; 95,846,751-102,501,812 bp).


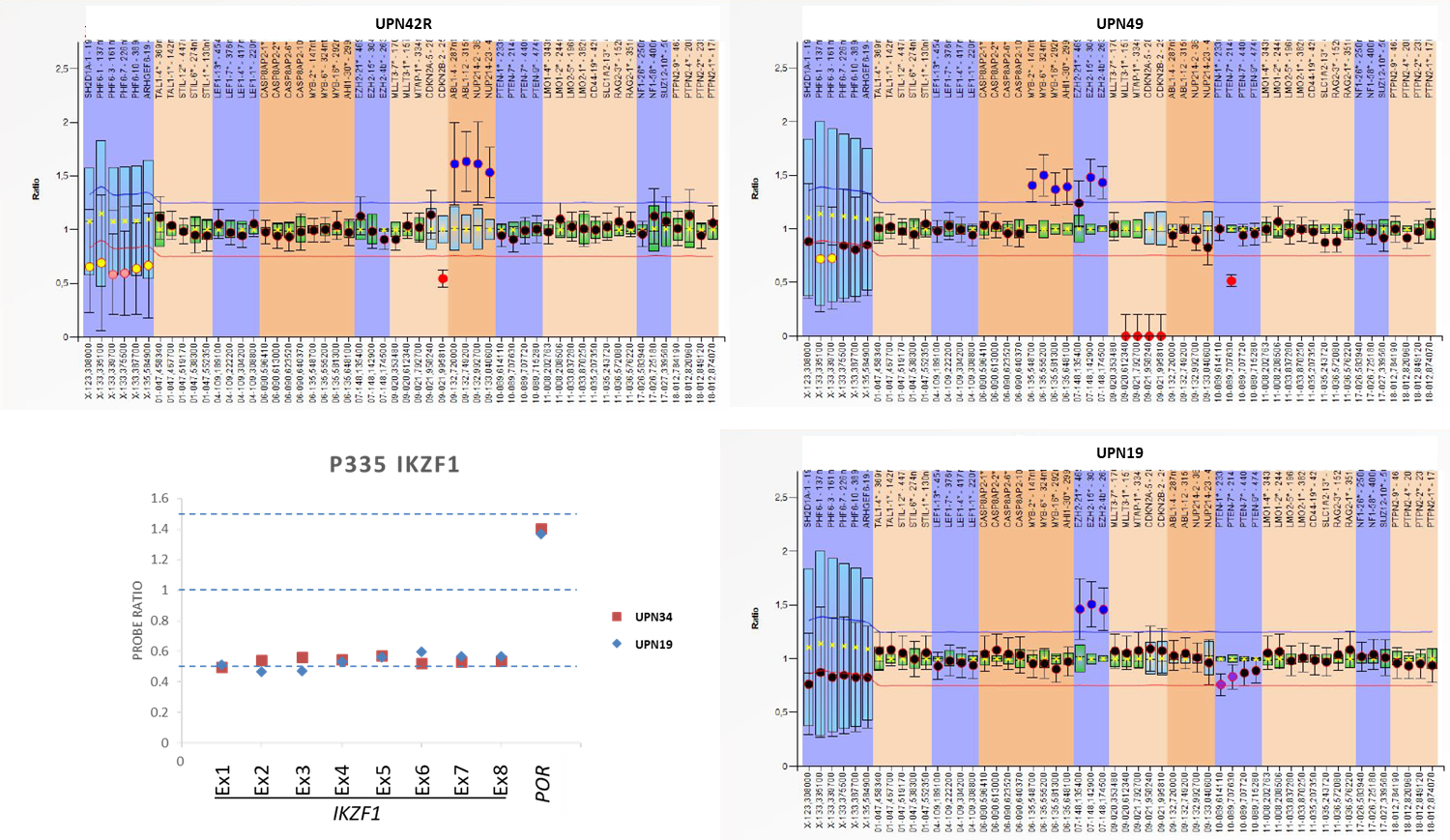


**Fig S6.** MLPA analyses of isochromosome 7q. MLPA analysis of UPN42R, 49 and 19 using the P383 T-ALL kit visualizing copy number changes in *EZH2* (7q36.1). MLPA analysis of UPN19 and 34 using the P335 T-ALL kit summarizing copy number changes in *IKZF1* (7p12.2) and *POR* (7q11.23).


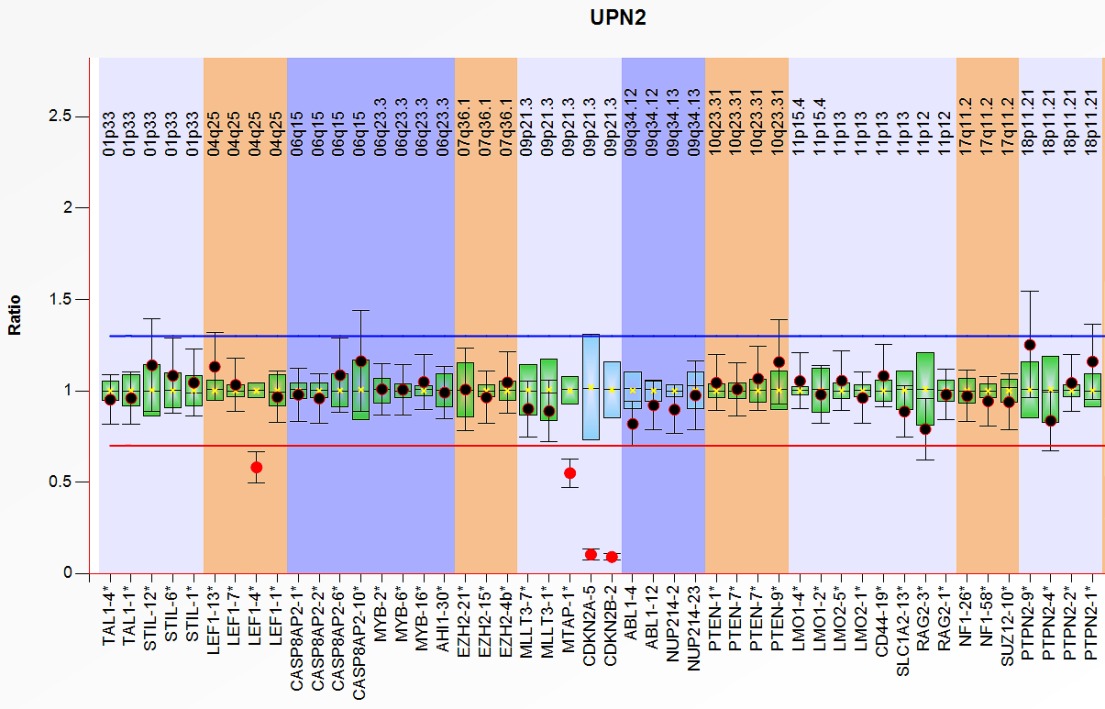


**Fig S7.** MLPA analysis of UPN2 sample. The results indicated a hemizygous loss of *LEF1* exon 4 and *MTAP*, as well as homozygous loss of *CDKN2A/B*. The deletions in *MTAP* and *CDKNA2A/B* confirmed the copy number data.


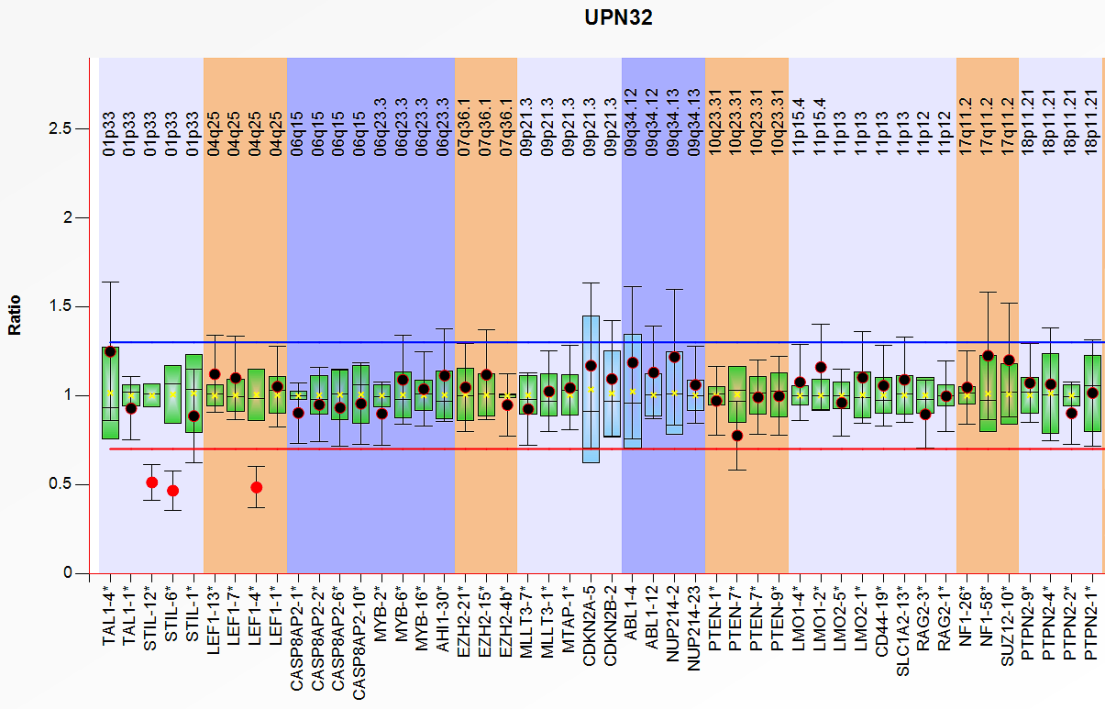


**Fig S8.** MLPA analysis of UPN32. Data analyses indicated hemizygous loss of *LEF1* exon 4 and *STIL*.


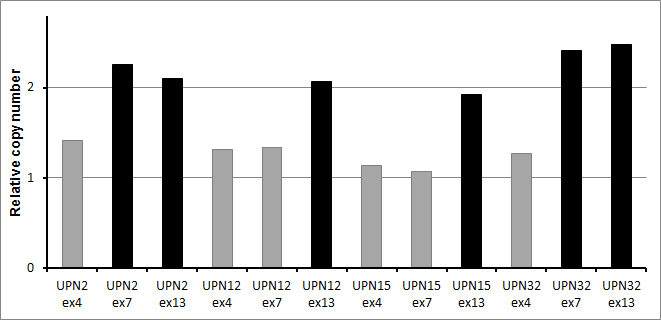


**Fig S9.** Copy number quantitative real-time PCR (copy number qPCR) of *LEF1*. Analyses for exon 4, 7 and 13 confirmed hemizygous loss of exon 4 in both cases UPN2 and UPN32.


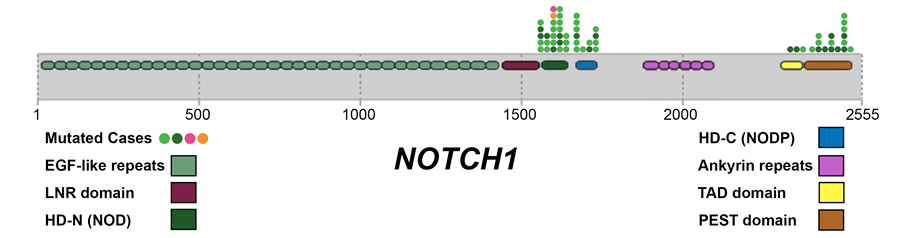


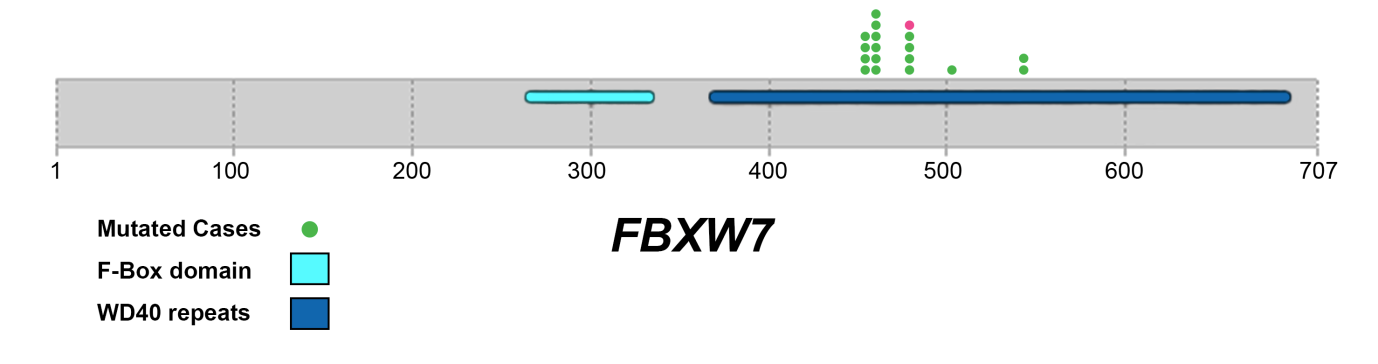


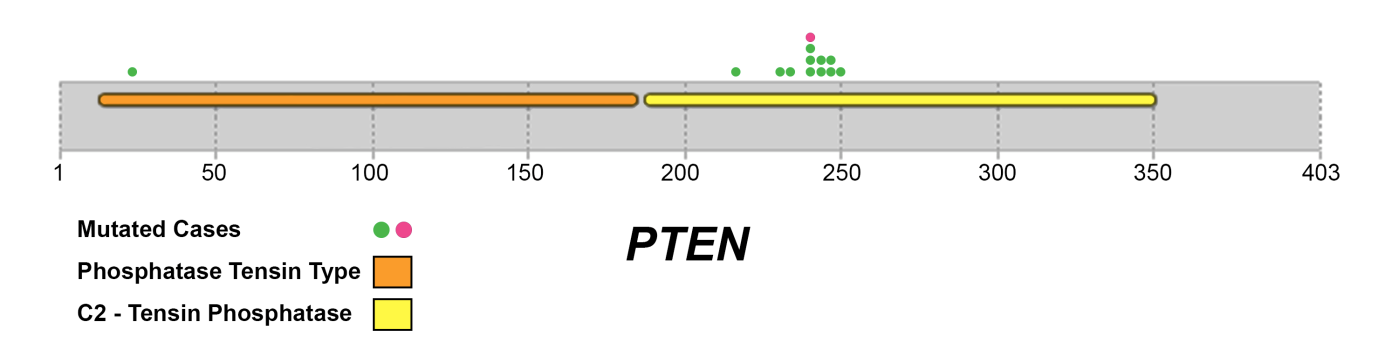


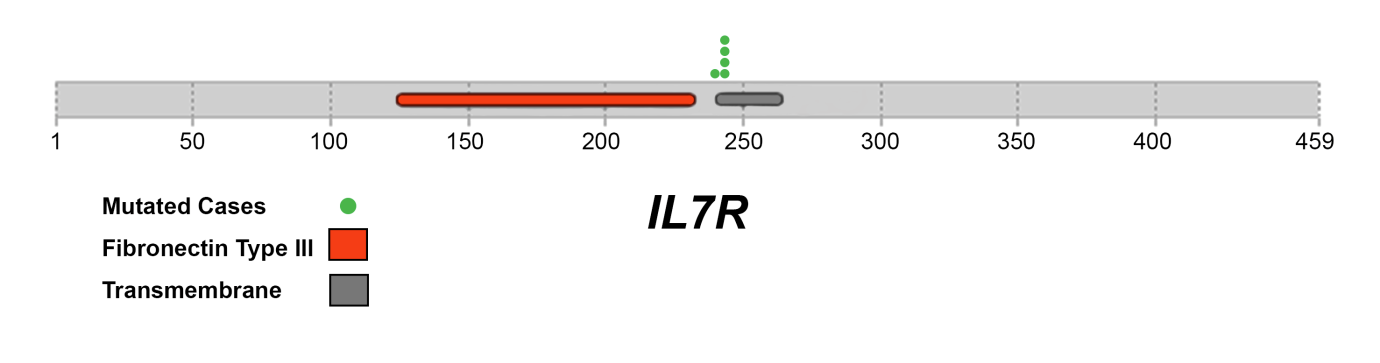


**Fig S10.** The incidence and location of *NOTCH1*, *FBXW7*, *PTEN* and *IL7R* mutations in TYA T-ALL patients. The green dots represent cases at diagnosis; pink dots are first relapse cases and the orange dot is a second relapse.


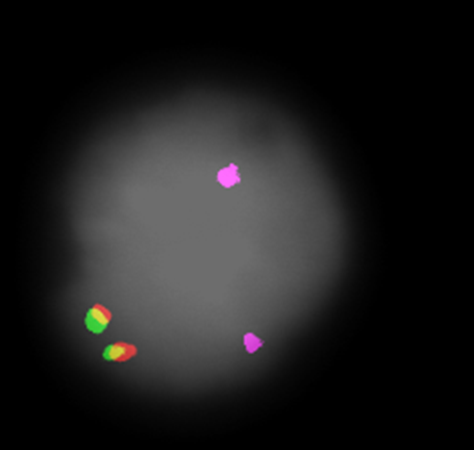


**Fig S11.** FISH analysis in UPN6. The analyses confirmed the absence of *CDKN2A* (biotin-Cy5, colored pink in the picture) deletion in UPN 6, with normal constellation of *TLX1* fusion probes (spectrum green/ spectrum red, yellow for fused signals).


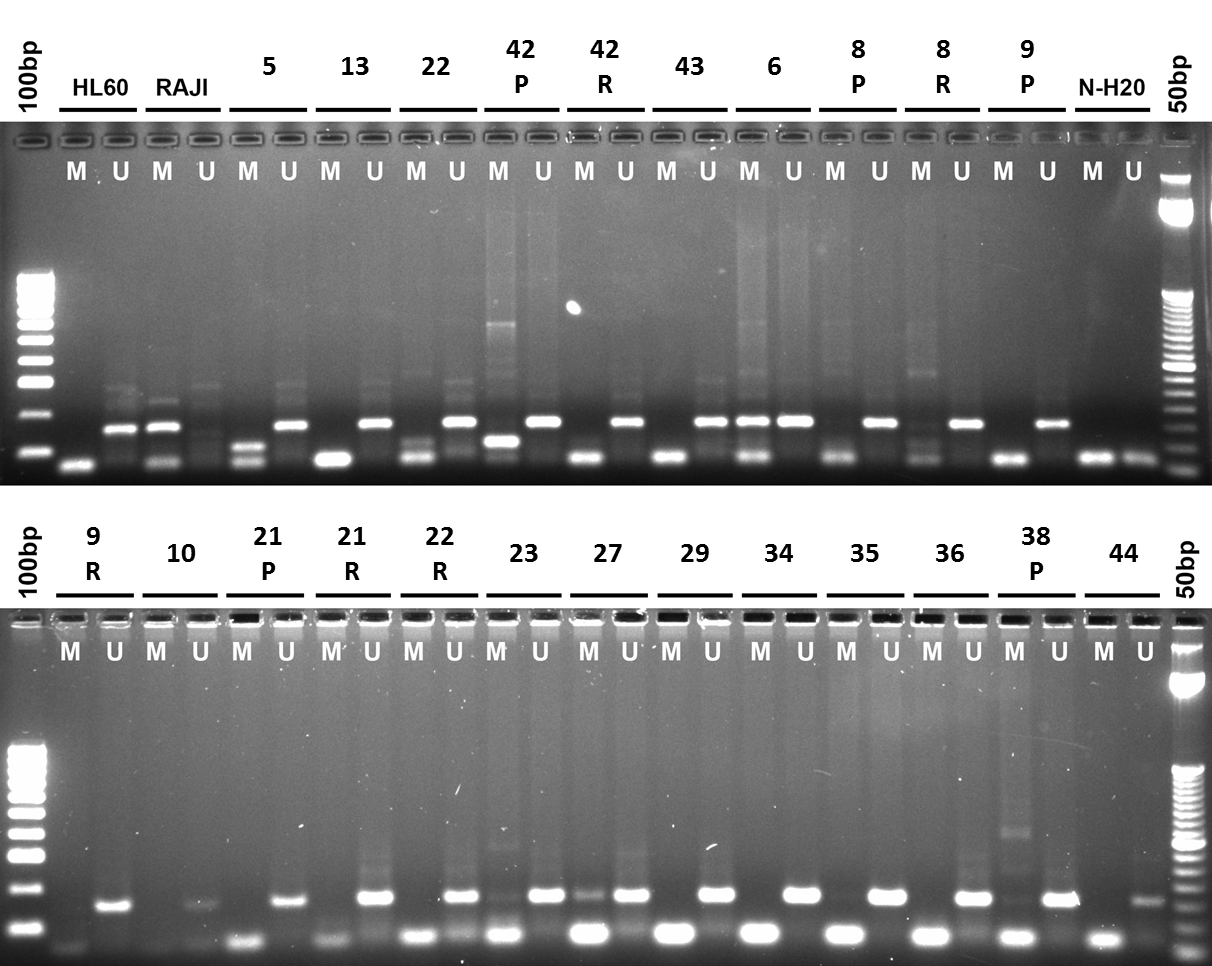


**Fig S12.** *CDKN2A* Methylation specific PCR (MSP) in 23 patient samples and 2 control cell lines**.** 100bp and 50bp ladders (Bioline) were used. M, methylated, U, unmethylated. N-H2O, no DNA control. The DNA from the HL60 cell line generated a strong band with unmethylated primers, whereas the DNA from the RAJI cell line produced a strong band with methylated primers. UPN6 demonstrated both methylated and unmethylated gene promoter, whereas all other samples demonstrated unmethylated gene promoters only.


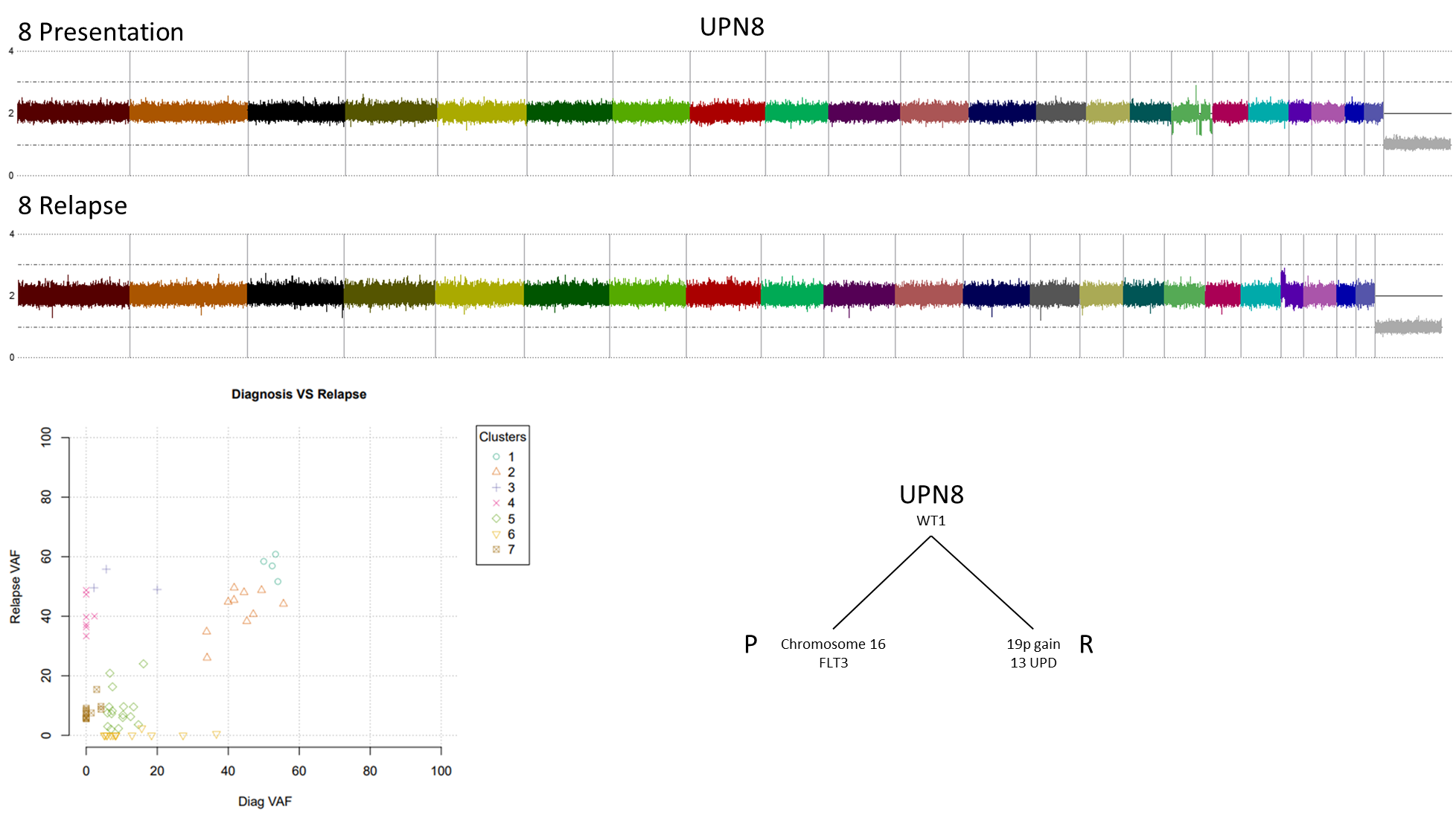


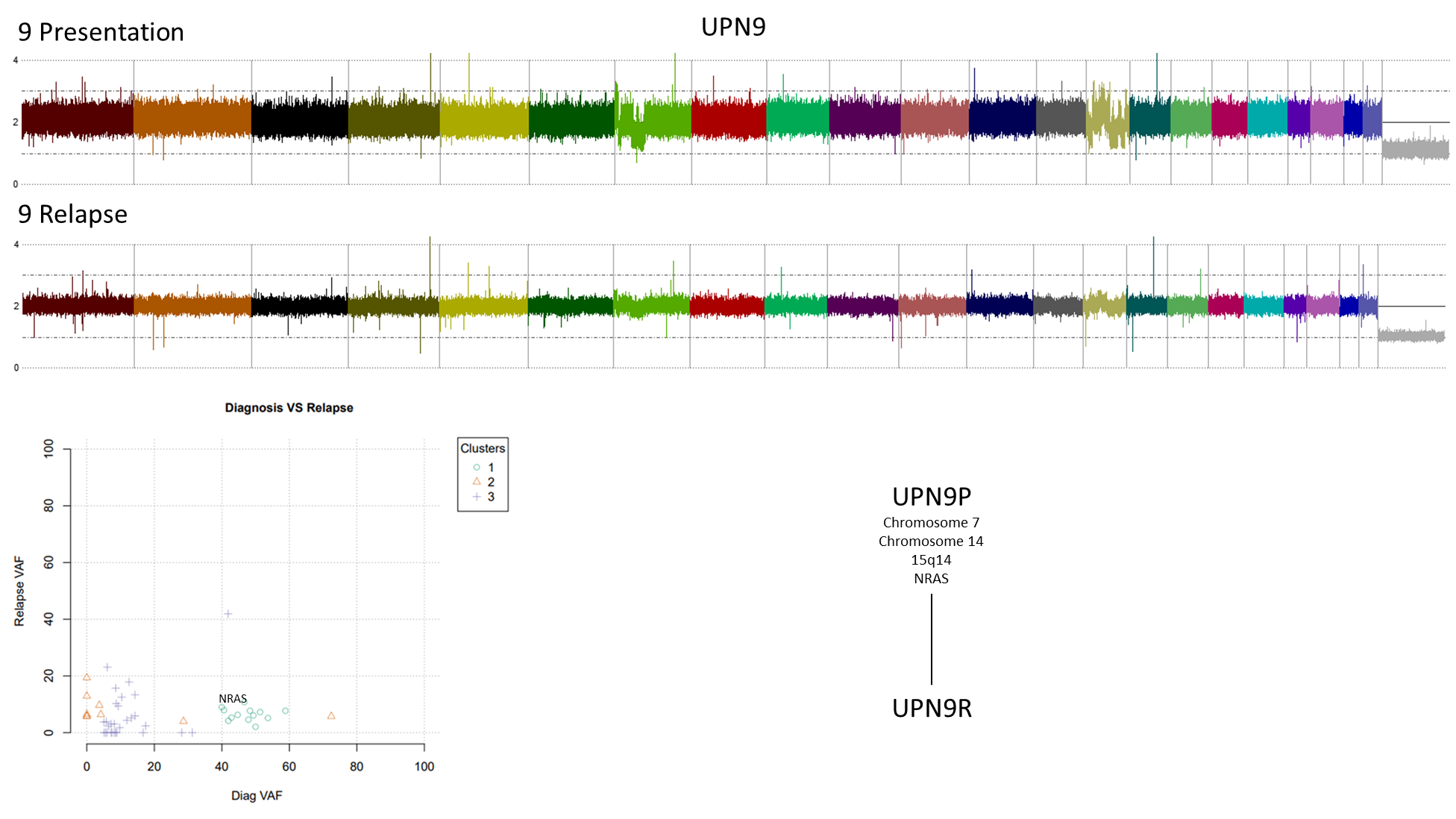


**
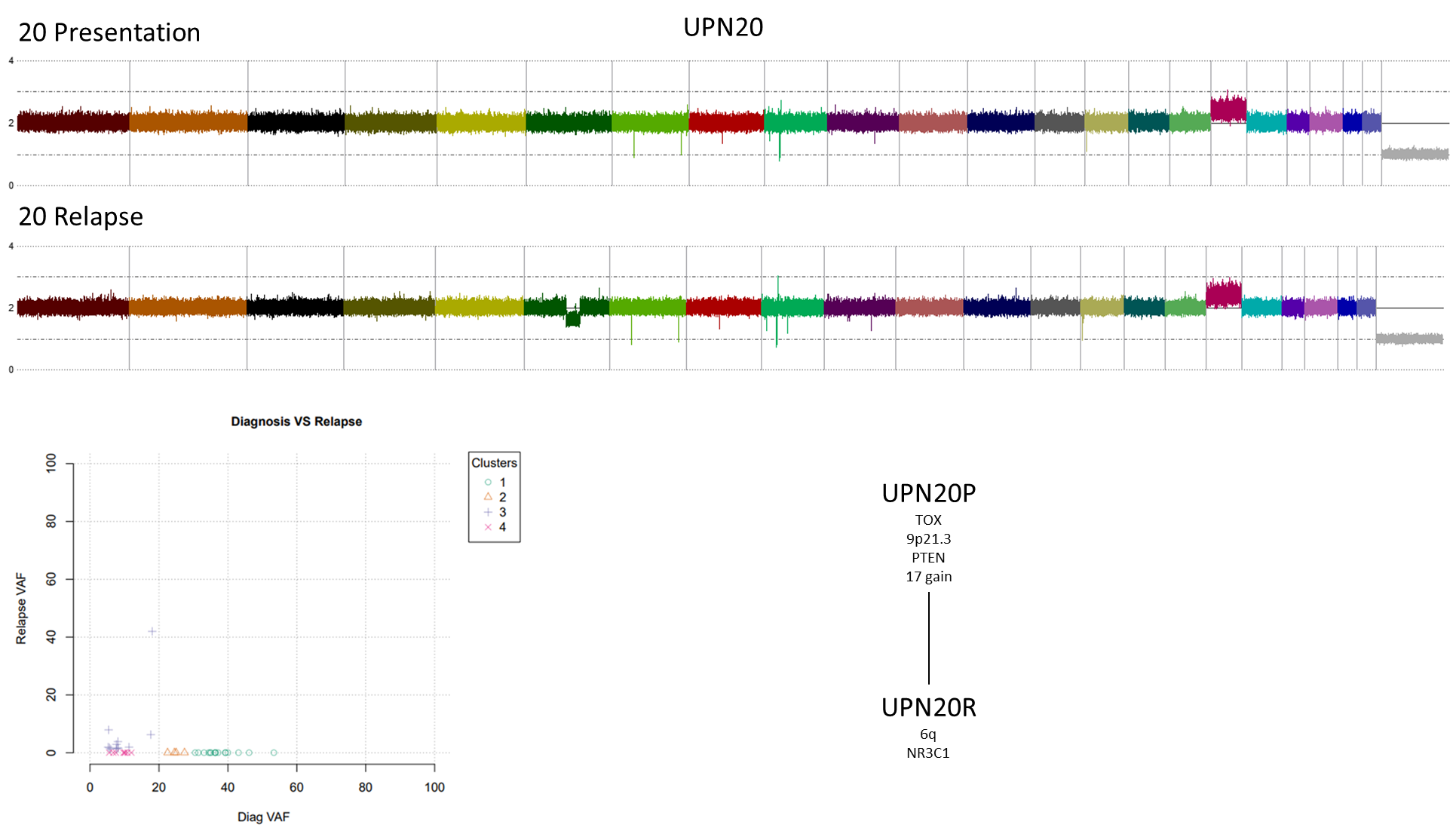
**


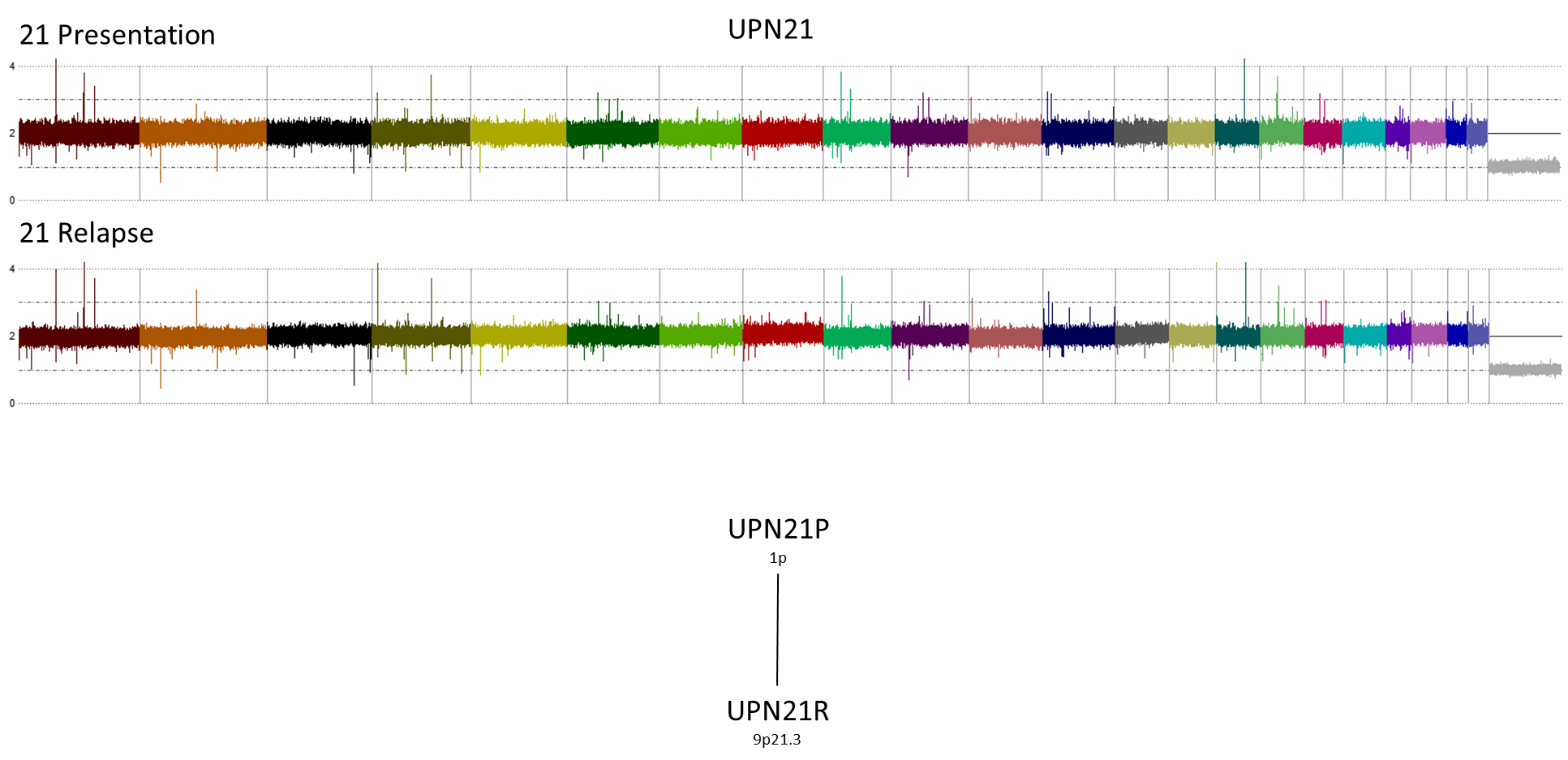


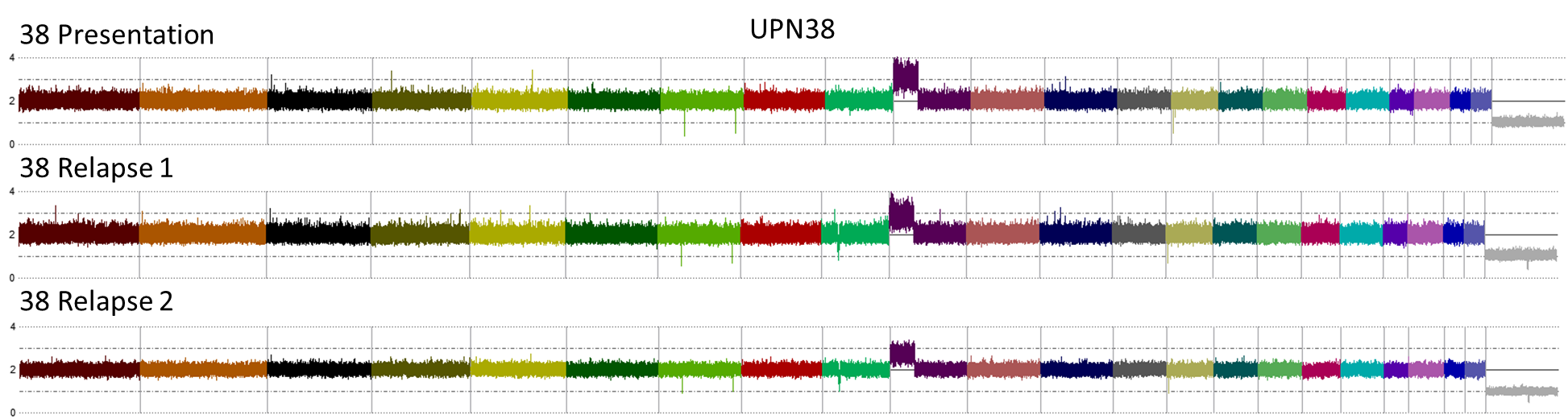


**
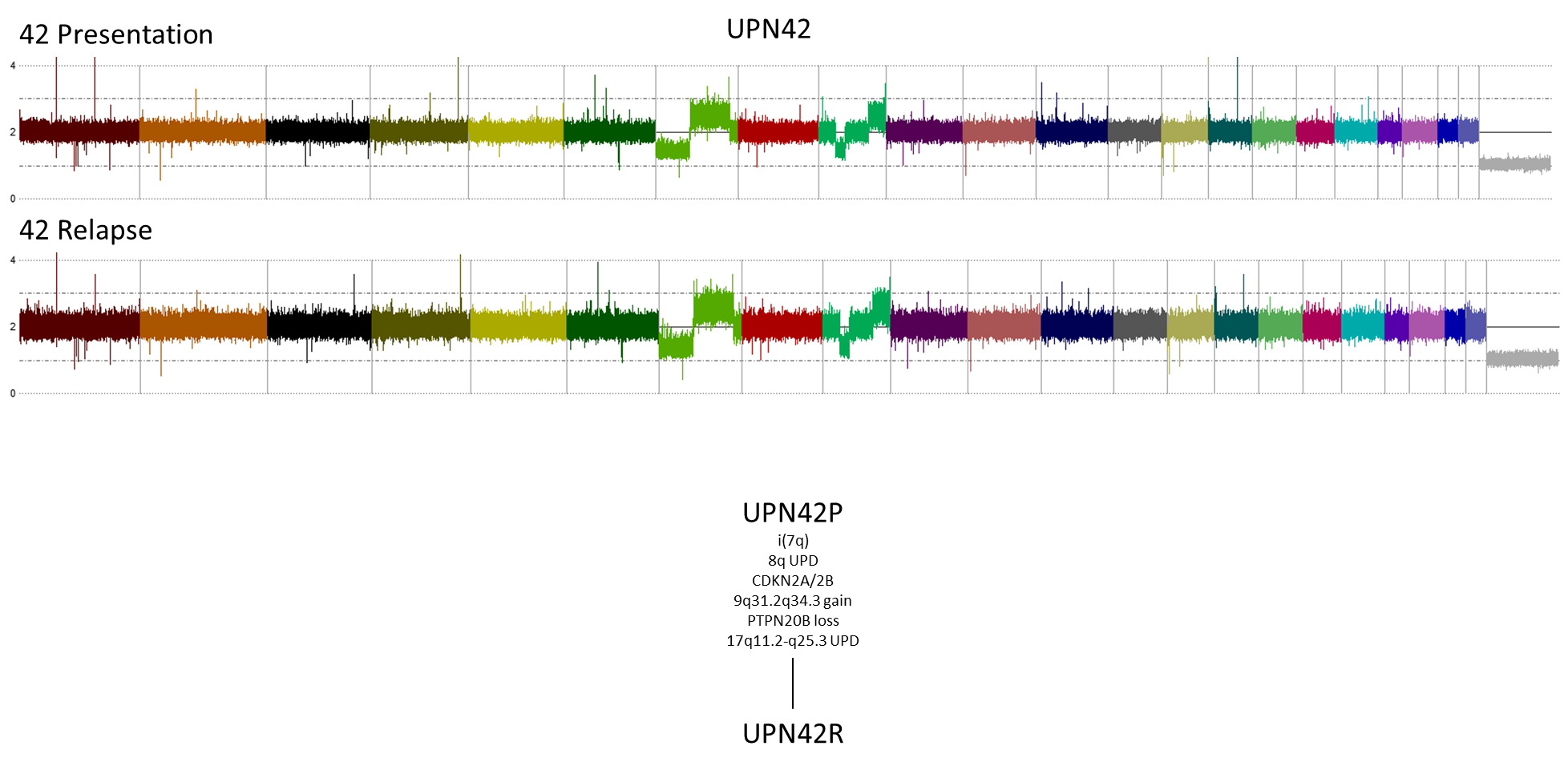
**

**Fig S13.** Comparative genetic analysis in matched presentation and relapse samples. Genome wide copy number analysis from chromosome 1 to chromosome X. VAF of missense mutations in presentation (P) and relapse (R). Phylogenetic tree depicting shared and acquired gene mutations.

**Fig S14.** Reverse transcription quantitative real-time PCR (RT-qPCR) data for *TLX1* gene expression using *B_2_M* as reference gene in TYA T-ALL. The analysis suggested *TLX1* aberrant upregulation in UPN 6, 13, 31, 18, 25 represented by the red bars. The presence of *TLX1* rearrangement, which results in *TLX1* overexpression, was confirmed by FISH analysis.


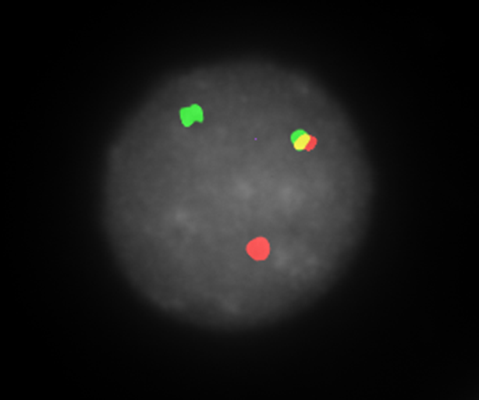


**Fig S15.** FISH confirmation of *TLX1* rearrangement in UPN 13. *TLX1* rearrangement leads to separation of the break apart probes (spectrum green/spectrum red), which under normal circumstances co-localize and generate a yellow signal.

**Fig S16.** *TLX3* gene expression in TYA T-ALL determined through reverse transcription quantitative real-time PCR (RT-qPCR) using *B_2_M* as reference gene. The analysis suggested aberrant upregulation in UPN 7 and 30 represented by the red bars. *TLX3* rearrangement, which results in *TLX3* overexpression, was confirmed by FISH analysis for UPN7 case.


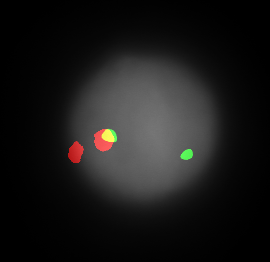


**Fig S17.** FISH confirmation of *TLX3* rearrangement in UPN 7 using in-house probes. *TLX3* rearrangement leads to separation of the break apart probes (spectrum green/spectrum red), which under normal circumstances co-localize and generate a yellow signal.


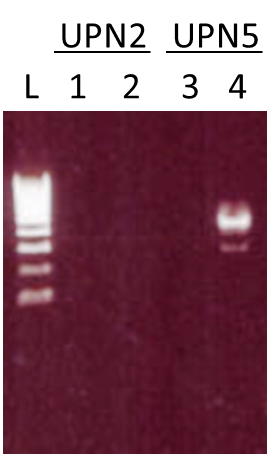


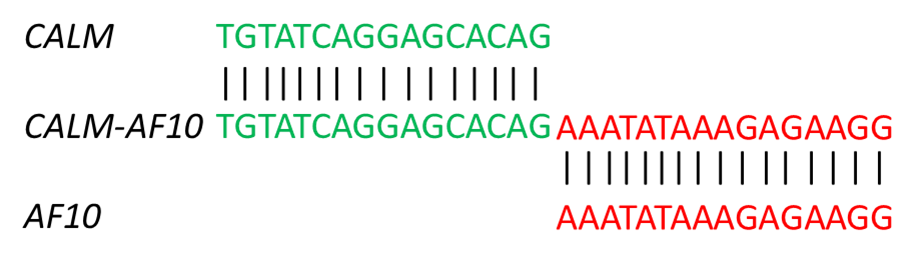


**Fig S18.** PCR analysis and Sanger sequencing in CC117 with *CALM-AF10* fusion. cDNA from patients UPN 2 and 5 was screened with *CALM* S1770 forward primer and *AF10* AS 559 and 1002 reverse primers. The large PCR product generated in UPN5 (lane 4) was subjected to Sanger sequencing and confirmed the presence of a fusion sequence between *CALM* (green) and *AF10* (red) genes. L, 100bp ladder (Bioline).

**References**

1. van Delft FW, Horsley S, Colman S, et al. Clonal origins of relapse in ETV6-RUNX1 acute lymphoblastic leukemia. *Blood.* 2011;117(23):6247-6254.

2. Nannya Y, Sanada M, Nakazaki K, et al. A robust algorithm for copy number detection using high-density oligonucleotide single nucleotide polymorphism genotyping arrays. *Cancer research.* 2005;65(14):6071-6079.

3. Mansur MB, Hassan R, Barbosa TC, et al. Impact of complex NOTCH1 mutations on survival in paediatric T-cell leukaemia. *BMC cancer.* 2012;12:9.

4. Weng AP, Ferrando AA, Lee W, et al. Activating mutations of NOTCH1 in human T cell acute lymphoblastic leukemia. *Science.* 2004;306(5694):269-271.

5. Kox C, Zimmermann M, Stanulla M, et al. The favorable effect of activating NOTCH1 receptor mutations on long-term outcome in T-ALL patients treated on the ALL-BFM 2000 protocol can be separated from FBXW7 loss of function. *Leukemia.* 2010;24(12):2005-2013.

6. Jotta PY, Ganazza MA, Silva A, et al. Negative prognostic impact of PTEN mutation in pediatric T-cell acute lymphoblastic leukemia. *Leukemia.* 2010;24(1):239-242.

7. Zenatti PP, Ribeiro D, Li W, et al. Oncogenic IL7R gain-of-function mutations in childhood T-cell acute lymphoblastic leukemia. *Nature genetics.* 2011;43(10):932-939.

8. Clappier E, Collette S, Grardel N, et al. NOTCH1 and FBXW7 mutations have a favorable impact on early response to treatment, but not on outcome, in children with T-cell acute lymphoblastic leukemia (T-ALL) treated on EORTC trials 58881 and 58951. *Leukemia.* 2010;24(12):2023-2031.

9. Case M, Matheson E, Minto L, et al. Mutation of genes affecting the RAS pathway is common in childhood acute lymphoblastic leukemia. *Cancer research.* 2008;68(16):6803-6809.

10. Gonzalez D, Martinez P, Wade R, et al. Mutational status of the TP53 gene as a predictor of response and survival in patients with chronic lymphocytic leukemia: results from the LRF CLL4 trial. *Journal of clinical oncology : official journal of the American Society of Clinical Oncology.* 2011;29(16):2223-2229.

11. Kearney L, Colman S. Specialized fluorescence in situ hybridization (FISH) techniques for leukaemia research. *Methods in molecular biology.* 2009;538:57-70.

12. Bateman CM, Colman SM, Chaplin T, et al. Acquisition of genome-wide copy number alterations in monozygotic twins with acute lymphoblastic leukemia. *Blood.* 2010;115(17):3553-3558.

13. Anderson K, Lutz C, van Delft FW, et al. Genetic variegation of clonal architecture and propagating cells in leukaemia. *Nature.* 2011;469(7330):356-361.

14. Herman JG, Graff JR, Myohanen S, Nelkin BD, Baylin SB. Methylation-specific PCR: a novel PCR assay for methylation status of CpG islands. *Proceedings of the National Academy of Sciences of the United States of America.* 1996;93(18):9821-9826.

15. Sulong S, Moorman AV, Irving JA, et al. A comprehensive analysis of the CDKN2A gene in childhood acute lymphoblastic leukemia reveals genomic deletion, copy number neutral loss of heterozygosity, and association with specific cytogenetic subgroups. *Blood.* 2009;113(1):100-107.

16. Breit TM, Mol EJ, Wolvers-Tettero IL, Ludwig WD, van Wering ER, van Dongen JJ. Site-specific deletions involving the tal-1 and sil genes are restricted to cells of the T cell receptor alpha/beta lineage: T cell receptor delta gene deletion mechanism affects multiple genes. *The Journal of experimental medicine.* 1993;177(4):965-977.

17. Van Vlierberghe P, van Grotel M, Tchinda J, et al. The recurrent SET-NUP214 fusion as a new HOXA activation mechanism in pediatric T-cell acute lymphoblastic leukemia. *Blood.* 2008;111(9):4668-4680.

18. Asnafi V, Radford-Weiss I, Dastugue N, et al. CALM-AF10 is a common fusion transcript in T-ALL and is specific to the TCRgammadelta lineage. *Blood.* 2003;102(3):1000-1006.

19. Li H, Durbin R. Fast and accurate short read alignment with Burrows-Wheeler transform. *Bioinformatics.* 2009;25(14):1754-1760.

20. McKenna A, Hanna M, Banks E, et al. The Genome Analysis Toolkit: a MapReduce framework for analyzing next-generation DNA sequencing data. *Genome research.* 2010;20(9):1297-1303.

21. DePristo MA, Banks E, Poplin R, et al. A framework for variation discovery and genotyping using next-generation DNA sequencing data. *Nature genetics.* 2011;43(5):491-498.

22. Van der Auwera GA, Carneiro MO, Hartl C, et al. From FastQ data to high confidence variant calls: the Genome Analysis Toolkit best practices pipeline. *Current protocols in bioinformatics / editoral board, Andreas D Baxevanis [et al].* 2013;11(1110):11 10 11-11 10 33.

23. Cibulskis K, Lawrence MS, Carter SL, et al. Sensitive detection of somatic point mutations in impure and heterogeneous cancer samples. *Nature biotechnology.* 2013;31(3):213-219.

24. Koboldt DC, Zhang Q, Larson DE, et al. VarScan 2: somatic mutation and copy number alteration discovery in cancer by exome sequencing. *Genome research.* 2012;22(3):568-576.

25. McLaren W, Pritchard B, Rios D, Chen Y, Flicek P, Cunningham F. Deriving the consequences of genomic variants with the Ensembl API and SNP Effect Predictor. *Bioinformatics.* 2010;26(16):2069-2070.

26. Miller CA, White BS, Dees ND, et al. SciClone: inferring clonal architecture and tracking the spatial and temporal patterns of tumor evolution. *PLoS Comput Biol.* 2014;10(8):e1003665.

27. Dang HX, White BS, Foltz SM, et al. ClonEvol: clonal ordering and visualization in cancer sequencing. *Ann Oncol.* 2017;28(12):3076-3082.

28. Miller CA, McMichael J, Dang HX, et al. Visualizing tumor evolution with the fishplot package for R. *BMC Genomics.* 2016;17(1):880.
